# Diversity and distribution of nearshore barnacle cyprids in southern California through the 2015–16 El Niño

**DOI:** 10.7717/peerj.7186

**Published:** 2019-07-01

**Authors:** Malloree L. Hagerty, Nathalie Reyns, Jesús Pineda, Annette F. Govindarajan

**Affiliations:** 1 Environmental and Ocean Sciences Department, University of San Diego, San Diego, CA, USA; 2 Biology Department, Woods Hole Oceanographic Institution, Woods Hole, MA, USA

**Keywords:** Barnacle cyprids, *Chthamalus fissus*, DNA barcoding, El Niño, Larvae, Species diversity

## Abstract

Abundance, species diversity, and horizontal distributions of barnacle cyprids offshore of La Jolla, southern California were described from May 2014 to August 2016 to determine how the nearshore barnacle larval assemblage changed before, during, and after the 2015–16 El Niño. The entire water column was sampled at five stations located within one km of shore with water depths of 4, 6, 8, 10, and 12 m during 33 cruises that encompassed the time when El Niño conditions impacted the area. Nearshore temperature and thermal stratification was concurrently measured using a CTD. Six identified cyprid species, including *Chthamalus fissus*, *Pollicipes polymerus*, *Megabalanus rosa*, *Tetraclita rubescens*, *Balanus glandula*, and *B. trigonus*, along with four unknown species, were collected in our samples. DNA barcoding was used to confirm identifications in a subset of the larvae. *C. fissus* was more than eight times more abundant than any other species, and while abundance varied by species, cyprid density was highest for all species except for *M. rosa* before and after the El Niño event, and lower during the environmental disturbance. There were significant differences in cross-shore distributions among cyprid species, with some located farther offshore than others, along with variability in cross-shore distributions by season. *C. fissus* cyprids were closest to shore during spring-summer cruises when waters were the most thermally stratified, which supports previous findings that *C. fissus* cyprids are constrained nearshore when thermal stratification is high. Relative species proportions varied throughout the study, but there was no obvious change in species assemblage or richness associated with El Niño. We speculate that barnacle cyprid species diversity did not increase at our study site during the 2015–16 El Niño, as it has in other areas during previous El Niño Southern Oscillation events, due to the lack of anomalous northward flow throughout the 2015–16 event.

## Introduction

The warm phase of El Niño Southern Oscillation (ENSO) in the Eastern Pacific is associated with widespread oceanic disturbance, as both environmental and advective processes can change during El Niño events. This in turn may affect marine communities, especially planktonic organisms that do not swim effectively against currents and are thus subject to changes in ocean circulation (Ohman et al., 2018). Southern California experienced El Niño conditions from April 2015 to May 2016 (McClatchie et al., 2016), and barnacle larval abundance in nearshore waters off La Jolla, southern California was lower during El Niño than it was before or after the disturbance (Hagerty, Reyns & Pineda, 2018; Pineda, Reyns & Lentz, 2018). The 1982–83 El Niño also impacted southern California, and two barnacle species usually found farther south appeared offshore of San Diego: *Megabalanus coccopoma* and *M. californicus* (Newman & McConnaughey, 1987). The 1997–98 ENSO event coincided with enhanced larval recruitment of *Balanus glandula*, *Chthamalus fissus*, and *C. dalli* barnacles in central and northern California (Connolly & Roughgarden, 1999). Similarly, coastal abundances of crab zoeae increased markedly in California during springs of El Niño years, due to northward transport from Baja California waters (Lynn & Bograd, 2002; Pérez-Brunius, López & Pineda, 2006), and the arrival of rare copepods increased species richness during 1997–98 and 2015–16 El Niño conditions (McClatchie et al., 2016).

Non-planktonic organisms have experienced similar fluctuations in abundance and species composition associated with El Niño in southern California: forage fish numbers decreased and fish assemblages showed both an increase in southern species and a decrease in northern species during the 2015–16 ENSO event (McClatchie et al., 2016). Species evenness of tidepool fish in La Jolla and San Diego increased during El Niño in 1997–98, when abundance of the dominant species dwindled (Davis, 2000). Additionally, the strong 1997–98 El Niño corresponded with a northern range shift of eastern tropical Pacific fishes into southern California, and some Panamic fish species were recorded in California for the first time (Lea & Rosenblatt, 2000). The present study was conducted in and focuses on southern California, but El Niño affects marine ecosystems in many locations (Arntz & Tarazona, 1990; Coffroth, Lasker & Oliver, 1990; Warwick, Clarke & Suharsono, 1990; Carballo, Olabarria & Osuna, 2002; Navarrete et al., 2002; Garcia, Vieira & Winemiller, 2003).

To determine changes in the barnacle cyprid assemblage in nearshore waters of La Jolla, southern California throughout El Niño, we measured abundance and species diversity, and characterized the horizontal distribution of late-stage barnacle larvae, or cyprids, during 33 collection cruises from May 2014 to August 2016, encompassing the time when the study area experienced El Niño conditions. During each cruise, we sampled the entire water column at five stations roughly perpendicular to the coast and within one km of shore. Pineda, Reyns & Lentz (2018) found that abundance and settlement of *C. fissus* barnacle larvae decreased, thermal stratification was lower, and high-frequency internal waves were less energetic at our sampling site during the 2015–16 El Niño compared to after this period. When stratification is high, internal motions such as internal tidal bores that have the potential to transport larvae are more energetic (Pineda, 1999; Pineda & López, 2002). Hagerty, Reyns & Pineda (2018) found that *C. fissus* cyprids were constrained to shore when thermal stratification was high, but it is unclear if other species adhere to the same pattern.

Marine invertebrate larvae, and barnacle larvae in particular, can be extremely difficult to identify to species by morphology (Chen, Høeg & Chan, 2013; Wong et al., 2014). DNA barcoding is an effective complementary method for obtaining species identifications (Bucklin, Steinke & Blanco-Bercial, 2011). The barcoding process usually involves the extraction of genomic DNA from an organism, followed by amplification via PCR of a target barcode marker, and then sequencing. Resulting DNA sequences can be matched to reference sequences from previously identified individuals (Govindarajan et al., 2015). The mitochondrial COI gene is a barcode marker that can successfully distinguish between barnacle species (Chen, Høeg & Chan, 2013; Wong et al., 2014).

We hypothesized that larval barnacle species diversity would increase during the 2015–16 El Niño year due to advection from southern regions. Specifically, we addressed the following questions: What species of barnacle cyprids are present offshore of La Jolla? Do abundance and species diversity of barnacle cyprids in the area change during El Niño conditions? Is cross-shore distribution of cyprids species-specific? Because of the importance of identifying species-specific patterns, we used DNA barcoding to confirm our morphological larval identifications.

## Materials and Methods

### Sample collections

**Figure 1:**
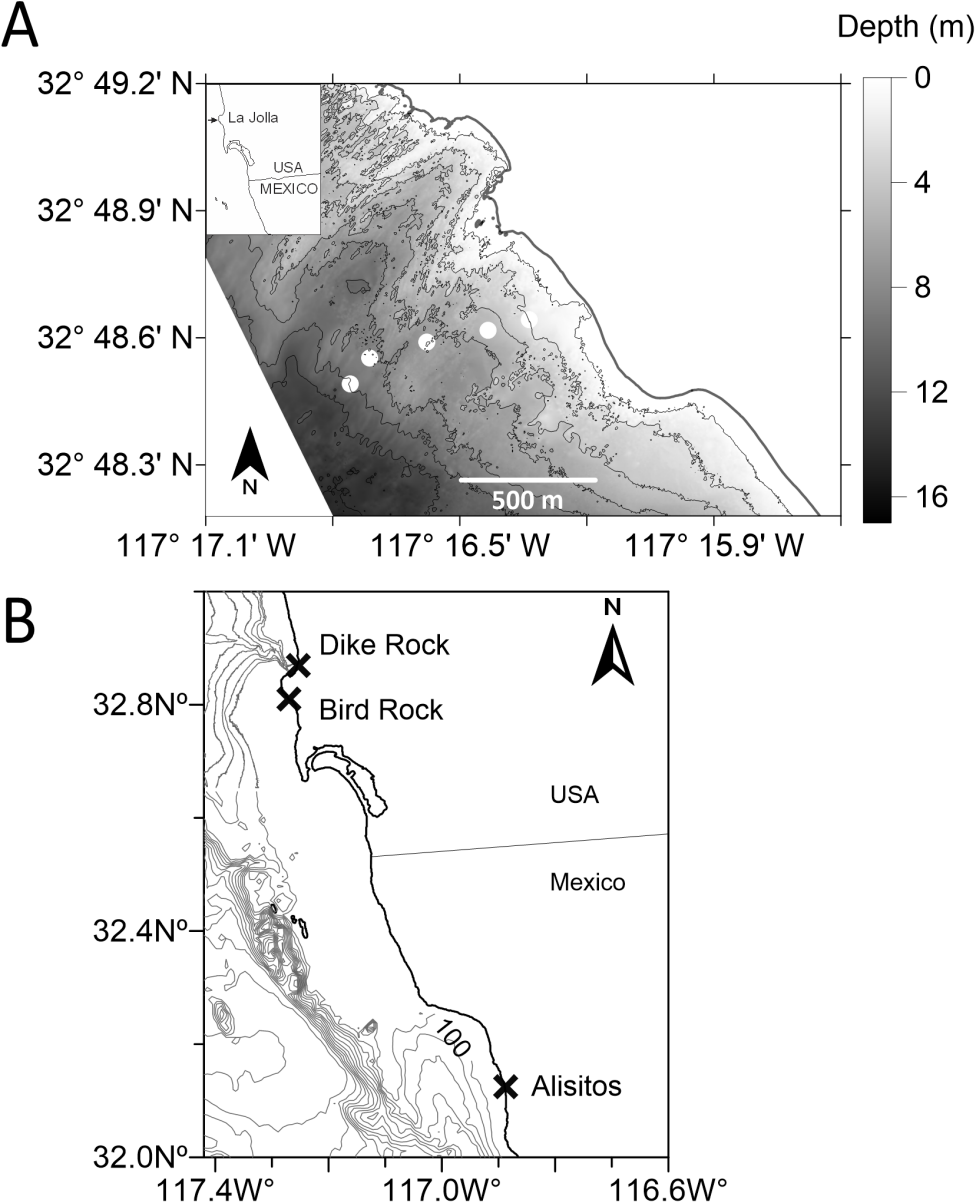
Study location. (A) Sampling site offshore of Bird Rock, La Jolla, southern California, USA. Circles indicate plankton sampling stations 280, 460, 640, 820, and 1,000 m from shore at a bottom depth of 4, 6, 8, 10, and 12 m, respectively, moving offshore from right to left. Bottom depth contours every two m relative to Mean Lower Low Water (MLLW), with the shallowest contour at MLLW. Lidar data from the 2013 NOAA Coastal California TopoBathy Merge Project, https://coast.noaa.gov/dataviewer/#/lidar/search/where:ID=2612. (B) Locations of adult barnacle collections (X): Bird Rock, La Jolla, California, USA; Dike Rock, La Jolla, California, USA; Alisitos, Baja California (Carignan et al., 2012).

All field collections were made under the California Department of Fish and Wildlife scientific collection permit SC-10523. Plankton samples were collected offshore of Bird Rock, La Jolla, California, USA and included five sampling stations at distances of 280, 460, 640, 820, and 1,000 m from shore at water depths of 4, 6, 8, 10, and 12 m, respectively (Fig. 1A). This study site was selected because of the large population of *C. fissus* barnacles on its shoreline, and because larvae of the predominant barnacle species in the area have been collected in surrounding waters (*C. fissus*, *B. glandula*, *Pollicipes polymerus*; Hoffman, 1989; Pineda, 1991, 1999; Pineda & López, 2002; Tapia & Pineda, 2007; Tapia et al., 2010). Plankton sampling was conducted on a 7.6 m boat, and consisted of 33 collection cruises: nine in spring-summer (May–July) and six in fall-winter (October–December) of 2014, eight in spring-summer (April–July) and four in fall-winter (October–December) of 2015, and six in summer (June–August) of 2016. All cruises were conducted during the day without consideration for lunar phase or tidal cycle. An Ebara 50DWXU6.4S Dominator submersible semivortex pump was used to filter two m^3^ of seawater in discrete two m depth bins from the surface to the bottom at each of the five sampling stations during each cruise. The pump was oscillated throughout the two m sampling intervals so that the entire water column was sampled at all stations. Each cruise resulted in 20 plankton samples unless sea state conditions or equipment failure interrupted sampling. Seawater was filtered through a 118-μm mesh net to capture barnacle cyprids, and plankton collected on the filter were immediately preserved in 100% ethanol.

Barnacle cyprids were enumerated and identified to species based on published morphological descriptions (Lewis, 1975; Brown & Roughgarden, 1985; Miller et al., 1989; Miller & Roughgarden, 1994; Shanks, 2001) using a dissecting microscope. Additionally, morphologically distinct cyprids were selected for DNA barcoding, preserved individually in ethanol, and refrigerated until molecular analysis was carried out. DNA barcoding was completed to ground truth morphological identifications and classify cyprids with unfamiliar morphology that we were otherwise unable to match to known species. After barcoding, morphological identifications were assessed, and cyprids that were unknown prior to barcoding were compared to reference sequences.

Morphologically distinct adult barnacles were collected from boulders in the intertidal at three locations to serve as a baseline for barnacle species in the area: onshore of the plankton sampling location at Bird Rock, La Jolla, California, USA; north of sampling location at Dike Rock, La Jolla, California, USA; south of sampling location at Alisitos, Baja California, Mexico (Fig. 1B). Collections were conducted on May 7, 2016 at Bird Rock, May 9, 2016 and June 20, 2016 at Dike Rock, and June 22, 2016 at Alisitos. The goal was to find ∼5 representative individuals for each different species, which resulted in 55 barnacles total. All adults were dissected; a small piece of tissue was removed, preserved in ethanol, and refrigerated until molecular analysis was carried out.

### Molecular analysis

Molecular analysis methods were slightly modified from the methods outlined by Govindarajan et al. (2015). For cyprids, the whole larva was placed into PCR tubes. For adults, Qiagen DNEasy Blood & Tissue extraction kits were used to extract DNA from the small piece of tissue removed during dissection. The mitochondrial COI gene was amplified using HCO2198 and LCO1490 primers (Folmer et al., 1994) or jgHCO2198 (Geller et al., 2013) and mlCOIintF primers (Leray et al., 2013). The PCR cycle for Folmer primer amplifications was: (a) an initial denaturation at 95 °C for 3 min; (b) 35 cycles of 95 °C 30 s, 48 °C 30 s, 72 °C 60 s; and (c) a final extension at 72 °C for 5 min. A touchdown protocol was used for amplifications with the Geller–Leray primers (Leray et al., 2013). PCR products were visualized with a 1% agarose gel stained with Biotium GelRed, and purified with Qiagen Qiaquick PCR Purification kits. After purification, DNA was qualified and quantified with a NanoDrop One Microvolume UV-Vis spectrophotometer and sequenced in both directions (Eurofins Scientific, Brussels, Belgium). Geneious 9.1.7 (Biomatters Limited, Auckland, New Zealand) was used to assemble and align chromatograms, and sequences were compared to reference sequences in GenBank using Basic Local Alignment Search Tool, accessed on 7 June 2017. All sequences generated in this study were submitted to GenBank (MK496547–MK496616).

### Diversity measurements

Species diversity was assessed for each cruise using Hurlbert’s (1971) modification of Sanders (1968) rarefaction index:

$$E\left( {{S_n}} \right) = \sum\limits_{i = 1}^s {\left[ {1 - {{\left( {\matrix{ {N - {N_i}} \cr n \cr } } \right)} \over {\left( {\matrix{ N \cr n \cr } } \right)}}} \right]} $$

where *E*(*S_n_*) is the expected number of species in a sample of *n* individuals selected at random from a collection containing *N* individuals, *S* species, and *N_i_* individuals in the *i*th species (Hurlbert, 1971). Rarefaction is used to compare sample collections of different sizes by reducing them to a common size (*n*), since species richness usually increases with *N* (Hurlbert, 1971). Mean *E*(*S*_20_) was calculated for each sampling period, and a randomization test (Manly, 1997) was completed to determine if differences in *E*(*S_n_*) values were significant.

### Cyprid distributions

Cyprid concentration (no. m^−3^) at each station was multiplied by station depth to standardize samples to cyprid density (no. m^−2^) to account for vertical dilution of larvae when comparing cyprid concentrations at stations of varying depths. Cyprid density across the five sampling stations for each species was averaged for each cruise. The mean distance from shore (MDS; Hagerty, Reyns & Pineda, 2018; referred to as “average distance offshore” in Shanks & Shearman, 2009) of each cyprid species for each cruise was calculated as:

$${\rm{MDS}} = {{\sum \left( {{\rm{No}}{\rm{.}}\;{{\rm{m}}^{ - 2}}\;{\rm{at}}\;{\rm{a}}\;{\rm{given}}\;{\rm{station}}\; \times \;{\rm{Distance}}\;{\rm{from}}\;{\rm{shore}}\;{\rm{of}}\;{\rm{station}}} \right)} \over {\sum \left( {{\rm{No}}{\rm{.}}\;{{\rm{m}}^{ - 2}}\;{\rm{at}}\;{\rm{all}}\;{\rm{stations}}} \right)}}$$

A one-way ANOVA was used to test for differences in MDS among cyprid species; ANOVA assumptions were met.

### Environmental variables

Temperature and depth measurements were recorded before and after sampling at each station with a SonTek CastAway-CTD. Mean temperature for each cruise was calculated as the average of all CTD temperature measurements, and thermal stratification was calculated for each station. Analysis of 1,251 CTD profiles indicated that thermal stratification is an excellent proxy for density stratification (top-to-bottom temperature difference is correlated with top-to-bottom density difference: *r* = 0.987). Furthermore, given the relatively widespread deployment of temperature loggers on moorings, our calculations allow comparisons of thermal stratification to be made with other studies that have collected temperature profile data (Hagerty, Reyns & Pineda, 2018; Pineda, Reyns & Lentz, 2018). Salinity was not considered because stratification at our study location is mainly dependent on vertical differences in temperature (Hagerty, 2017).

Thermal stratification was defined as change in temperature m^−1^ (ΔT °C m^−1^), as in Hagerty, Reyns & Pineda, 2018, and calculated as:

$${\rm{Thermal\; stratification}} = {{\left( {{\rm{Temperature\; at\; surface}} - {\rm{Temperature\; at\; bottom}}} \right)} \over {\left( {{\rm{Depth\; of\; bottom\; temperature}} - {\rm{Depth\; of\; surface\; temperature}}} \right)}}$$

Zonal stratification (Hagerty, Reyns & Pineda, 2018) was calculated by averaging thermal stratification at the three offshore stations–640, 820, and 1,000 m from shore with bottom depths of 8, 10, and 12 m, respectively—for each cruise. These stations were chosen to represent zonal thermal stratification because they were frequently associated with the highest stratification at our study site.

## Results

### Cyprid abundance and species diversity

Molecular analysis resulted in 70 successful sequences: 26 cyprids and 44 adults. In all cases, morphological identifications were affirmed by the barcoding results. We found that the Geller–Leray primer set (Geller et al., 2013; Leray et al., 2013) worked better for our samples than the Folmer set (Folmer et al., 1994). During the first round of barcoding, we started with the Folmer set and switched to the Geller–Leray set after achieving little success. A contributing factor may have been contamination with rose Bengal, as some near-bottom samples with heavy sediment content had been stained, and we discovered during our first round of barcoding that DNA from stained cyprids could not be amplified with our procedure (0 of 16 stained cyprids were successfully barcoded). Easton & Thistle (2014) successfully amplified and sequenced DNA from 122 copepods stained with rose Bengal using a Chelex®-based procedure, which is thought to remove any inhibitory compounds from samples. Of the 27 cyprids that did not come from rose Bengal samples during the first round of barcoding, Folmer primers were used for 13 and Geller–Leray primers were used for the remaining 14. DNA was successfully amplified and sequenced for five cyprids using the Geller–Leray method (36%), but only two cyprids were successfully barcoded using the Folmer method (15%). The Folmer primers were not used during the second round of barcoding, when 19 barcodes were obtained from 50 attempted cyprids (38% success rate using only Geller–Leray primer set). Table 1 outlines how many cyprids were barcoded from each sampling period.

**Table 1:** Number of cyprids on which barcoding was attempted, and number of successfully barcoded cyprids, from each sampling period.

Sampling period	No. attempted	No. barcoded
Spring-Summer 2014	27	4
Fall-Winter 2014	0	0
Spring-Summer 2015	46	13
Fall-Winter 2015	8	2
Summer 2016	11	7

**Figure 2:**
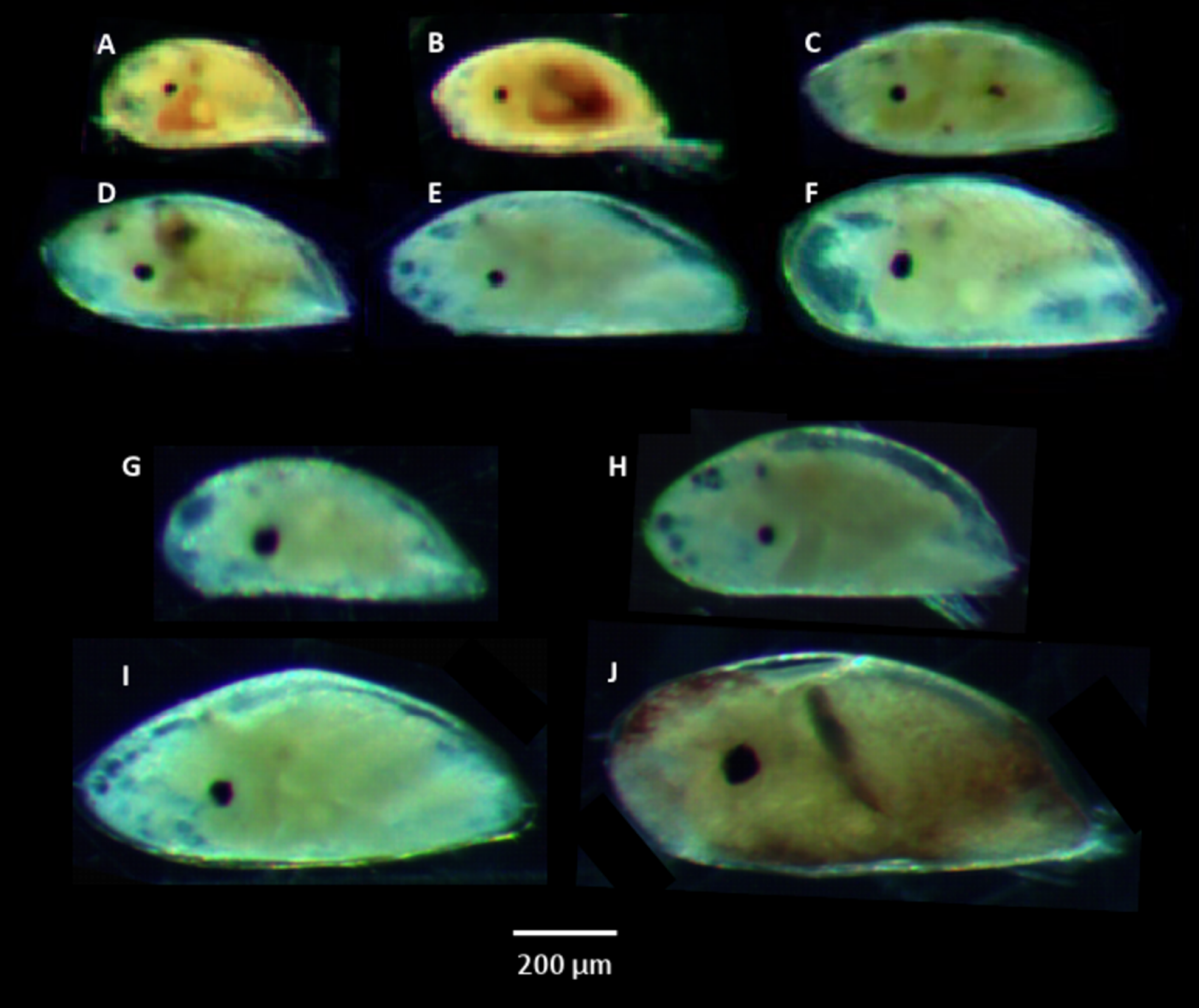
Cyprid species identified from plankton samples. (A) *Pollicipes polymerus*. (B) *Chthamalus fissus*. (C) *Balanus glandula*. (D) *Tetraclita rubescens*. (E) *Balanus trigonus*. (F) *Megabalanus rosa*. (G–J) four unknown species.

Six species of cyprids were identified, belonging to five genera and four families: *C. fissus*, *B. glandula*, *P. polymerus*, *Tetraclita rubescens*, *Megabalanus rosa*, and *B. trigonus* (Figs. 2A–2F). *Chthamalus* spp., *B. glandula*, and *P. polymerus* cyprids were identified morphologically, and were confirmed by molecular analysis (Table 2). *C. fissus* cyprids were given a tentative morphological identification of *Chthamalus* spp., because larvae of *C. fissus* and *C. dalli* are morphologically identical (Miller et al., 1989). However, *C. fissus* is the most abundant local barnacle and *C. dalli* is rare in San Diego, California (Morris, Abbott & Haderlie, 1980). Indeed, all of our barcoded *Chthamalus* cyprids and adults were identified as *C. fissus*. Three additional cyprid species, *T. rubescens*, *M. rosa*, and *B. trigonus*, were identified molecularly (Table 2). There were four distinct morphotypes of unknown cyprids, which were not successfully sequenced due to a failure to amplify their DNA, which could be due to low quality or insufficient quantity of DNA (Figs. 2G–2J). Six species of adults were identified, matching the identifications of the known cyprid species other than *B. trigonus*, and one additional species. Three adults—one collected at Dike Rock and two collected in Alisitos, Baja California—matched an unclassified *Sessilia*, *Balanoidea* sp. MM-2014 (GenBank: HG970516.1), collected from a soft coral in the genus *Leptogorgia* in Florida, USA (Malay & Michonneau, 2014).

**Table 2:** Tentative morphological identification, DNA sequence length, Basic Local Alignment Search Tool (BLAST) results (accessed 7 June 2017), and final species identification for barcoded cyprids.

Cyprid #—GenBank Accession #	Morphological ID	Sequence length (bp)	BLAST results		Final ID
			% Query coverage	% Identity	
1—MK496547	*Chthamalus* spp.	328	100	99	*Chthamalus fissus*
2—MK496548	*Chthamalus* spp.	313	100	98	*Chthamalus fissus*
3—MK496549	*Chthamalus* spp.	315	100	99	*Chthamalus fissus*
4—MK496550	*Chthamalus* spp.	581	100	100	*Chthamalus fissus*
5—MK496551	*Chthamalus* spp.	313	100	100	*Chthamalus fissus*
6—MK496552	*Chthamalus* spp.	313	100	99	*Chthamalus fissus*
7—MK496553	*Chthamalus* spp.	312	100	99	*Chthamalus fissus*
8—MK496554	*Chthamalus* spp.	310	100	100	*Chthamalus fissus*
9—MK496555	*Chthamalus* spp.	312	100	100	*Chthamalus fissus*
10—MK496556	*Chthamalus* spp.	311	100	100	*Chthamalus fissus*
11—MK496557	*Chthamalus* spp.	312	100	99	*Chthamalus fissus*
12—MK496558	*Balanus glandula*	468	100	100	*Balanus glandula*
13—MK496559	*Balanus glandula*	310	100	100	*Balanus glandula*
14—MK496560	*Balanus glandula*	273	100	100	*Balanus glandula*
15—MK496561	*Pollicipes polymerus*	307	100	100	*Pollicipes polymerus*
16—MK496562	Unknown A	306	100	100	*Tetraclita rubescens*
17—MK496563	Unknown A	238	90	100	*Tetraclita rubescens*
18—MK496564	Unknown A	306	100	100	*Tetraclita rubescens*
19—MK496565	Unknown A	306	100	100	*Tetraclita rubescens*
20—MK496566	Unknown A	307	100	100	*Tetraclita rubescens*
21—MK496567	Unknown A	291	100	100	*Tetraclita rubescens*
22—MK496568	Unknown B	306	100	99	*Megabalanus rosa*
23—MK496569	Unknown B	312	100	99	*Megabalanus rosa*
24—MK496570	Unknown B	311	100	99	*Megabalanus rosa*
25—MK496571	Unknown B	314	100	99	*Megabalanus rosa*
26—MK496572	Unknown C	278	100	99	*Balanus trigonus*

**Figure 3:**
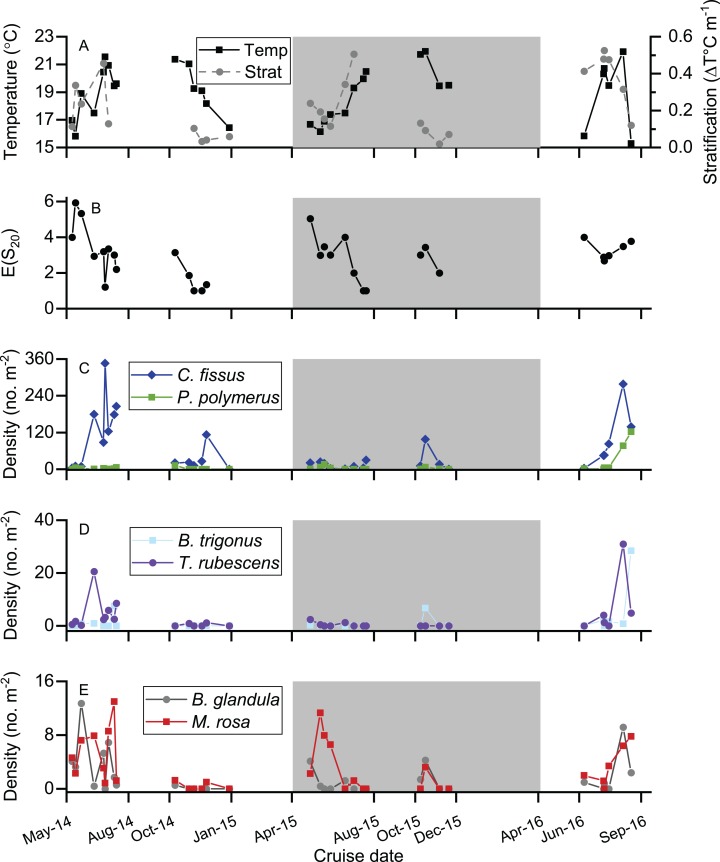
Time series of temperature, thermal stratification, species diversity, and cyprid density. (A) Water temperature and thermal stratification during each cruise. (B) Cyprid species diversity calculated for each cruise, where *E*(*S*_20_) is the expected number of species in a random sample of 20 individuals. (C–E) Density of each cyprid species during each cruise, separated into three sections due to the large range in densities across species. (C) *Chthamalus fissus* and *Pollicipes polymerus*. (D) *Tetraclita rubescens* and *Balanus trigonus*. (E) *Balanus glandula* and *Megabalanus rosa*. Shaded region represents the time period when ENSO conditions were impacting the study site.

**Figure 4:**
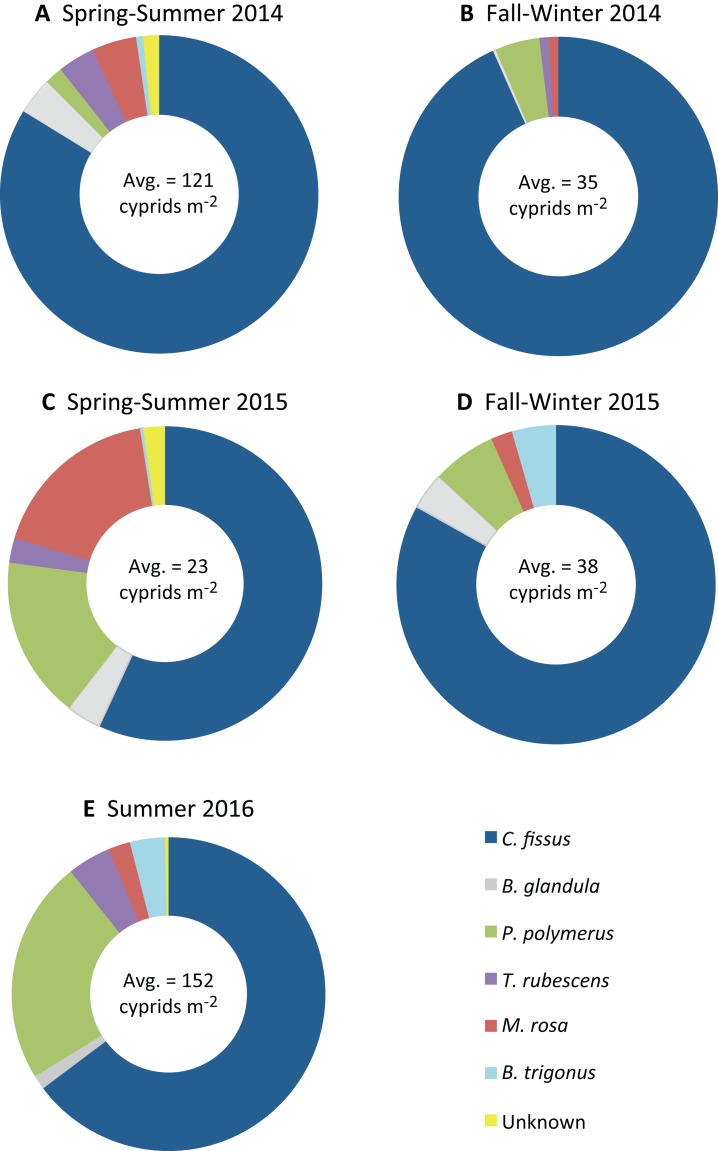
Proportion and average abundance of cyprid species. Relative proportion of total cyprids for each species (*Chthamalus fissus, Balanus glandula, Pollicipes polymerus, Tetraclita rubescens, Megabalanus rosa, Balanus trigonus*), and average cyprid abundance during sampling periods: (A) spring-summer 2014 (*N* = 149 samples), (B) fall-winter 2014 (*N* = 108 samples), (C) spring-summer 2015 (*N* = 136 samples), (D) fall-winter 2015 (*N* = 78 samples), (E) summer 2016 (*N* = 120 samples). ENSO conditions were impacting the area during spring-summer 2015 and fall-winter 2015 sampling periods.

Cyprid abundance varied by species, but was highest for most species in spring-summer 2014 and summer 2016 (Figs. 3C–3E). *P. polymerus* cyprids had relatively similar densities throughout all sampling periods until a large peak in density in summer 2016 (Fig. 3C). In contrast, *M. rosa* had higher relative abundances during spring-summer periods than during fall-winter periods (Fig. 3E). *C. fissus* was by far the most abundant cyprid found in samples (maximum density = 346 cyprids m^−2^), followed by *P. polymerus* (max. = 122 m^−2^) while *T. rubescens* (max. = 30 m^−2^), *B. trigonus* (max. = 28 m^−2^), *M. rosa* (max. = 12 m^−2^), and *B. glandula* (max. = 12 m^−2^) were found at low densities (Fig. 4). Cyprids were present in relatively large numbers starting in 2014 (average density = 121 cyprids m^−2^ in spring-summer 2014), decreased in fall-winter 2014 and remained low throughout 2015 (average density did not exceed 38 cyprids m^−2^ during these sampling periods), and increased again in summer 2016 (average density = 152 cyprids m^−2^). The highest density for the dominant species, *C. fissus*, and the two least abundant species, *B. glandula* and *M. rosa*, were collected during spring-summer 2014. The highest densities of *P. polymerus*, *T. rubescens*, and *B. trigonus* occurred in summer 2016 (Fig. 3).

All six identified cyprid species were collected during spring-summer of 2014 and 2015, as well as during the 2016 sampling period, but *B. trigonus* cyprids were not found in fall-winter of 2014, and *T. rubescens* cyprids were not collected in fall-winter of 2015 (Figs. 3 and 4). Species assemblage varied by sampling period, but *C. fissus* cyprids dominated the samples during all five periods (Fig. 4). *C. fissus* cyprids were collected during all cruises except December 29, 2014 and November 20, 2015, when no cyprids were found in our samples. The relative abundance of *C. fissus* was noticeably lower compared to other species during spring-summer 2015 and summer 2016, mainly due to an increase of *M. rosa* and *P. polymerus* cyprids (Fig. 4). Unknown cyprid species were not collected during fall-winter cruises in 2014 or 2015 (Fig. 4).

**Figure 5:**
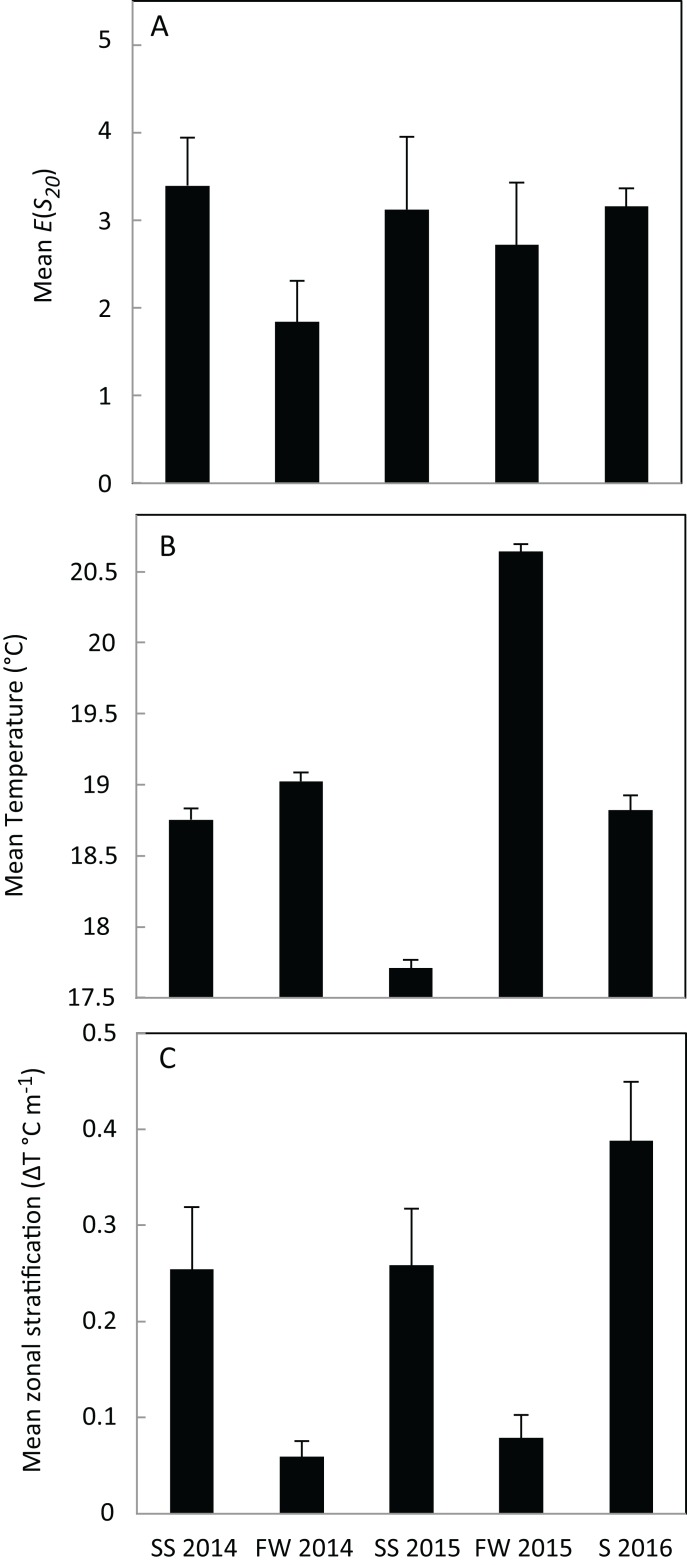
Mean species diversity and hydrographic conditions. (A) Mean species diversity, *E*(*S*_20_), for each sampling period: spring-summer 2014 (*N* = 9 cruises), fall-winter 2014 (*N* = 6 cruises), spring-summer 2015 (*N* = 8 cruises), fall-winter 2015 (*N* = 4 cruises), summer 2016 (*N* = 6 cruises). (B) Mean temperature and (C) mean zonal stratification for each sampling period. Error bars represent 1 SE. ENSO conditions were impacting the area during spring-summer 2015 and fall-winter 2015 sampling periods.

Species diversity was highest on May 14, 2014 (*E*(*S*_20_) = 5.92) and lowest on the 6th and 18th of November in 2014, and on the 16th and 20th of July in 2015, when *C. fissus* was the only species present (Fig. 3B). *E*(*S*_20_) measurements were more variable in spring-summer 2014 and spring-summer 2015 (ranging 1–5.92) than during the other sampling periods (Fig. 3B). Mean *E*(*S*_20_) was comparable for most sampling periods, except in fall-winter 2014, when it was low (Fig. 5).

We conducted a randomization analysis (Manly, 1997) to test for differences in *E*(*S*_20_) means among the five sampling periods: spring-summer 2014, fall-winter 2014, spring-summer 2015, fall-winter 2015, and summer 2016. Random samples of *n_ki_* values were taken without replacement from the vector comprising the entire series of *E*(*S*_20_) (*N* = 23), where *k* is sample period, and *i* is the number of *E*(*S*_20_) values in each sample period. That is, the 23 *E*(*S*_20_) values were randomly redistributed among the five sampling period groups. We then calculated the *F* one-factor ANOVA statistic, and this was repeated 10,000 times. The approximate *p*-value is the proportion of *F* values from the randomized data sets that are larger than the *F* computed from our data set; *p* = 0.3932. This indicates there were no significant differences in *E*(*S*_20_) among the sampling periods.

### Cyprid horizontal distributions and water column conditions

There were significant differences in MDS among cyprid species (ANOVA results: *F* = 15.13, *p* = 0.00016, d*f* = 1), and a Tukey post hoc test was completed for pairwise comparisons. *M. rosa* and *B. glandula* had significantly greater MDS values than *C. fissus*, meaning *M. rosa*, and *B. glandula* cyprids were distributed significantly farther from shore than *C. fissus* (*q_s_* = 225.17, *p* = 0.000043 and *q_s_* = 172.45, *p* = 0.0072, respectively). *M. rosa* was also significantly farther offshore than *P. polymerus* (*q_s_* = 168.51, *p* = 0.010). Average MDS for *C. fissus* cyprids was greater during fall-winter than spring-summer and summer sampling periods (Table 3), which means *C. fissus* cyprids were distributed closer to shore in spring-summer. In contrast, *P. polymerus* cyprids were closer to shore on average during fall-winter sampling periods (Table 3). While too few *M. rosa* cyprids were collected in fall-winter of 2015 to make comparisons, the average MDS value for *M. rosa* in fall-winter 2014 was lower than that of the spring-summer periods of 2014 and 2015, as well as that of summer 2016. Another notable result was that *T. rubescens* cyprids were located closer to shore in summer 2016 compared to earlier sampling periods (Table 3).

**Table 3:** MDS values for cyprid species. ENSO conditions were impacting the area during spring-summer 2015 and fall-winter 2015 sampling periods.

Average MDS ± SE	*Chthamalus fissus*	*Balanus glandula*	*Pollicipes polymerus*	*Tetraclita rubescens*	*Megabalanus rosa*	*Balanus trigonus*
SS 2014	538.08 ± 27.82	649.28 ± 49.56	587.89 ± 51.34	726.54 ± 130.80	746.99 ± 37.19	550.00 ± 51.96
FW 2014	613.03 ± 41.98	–	537.21 ± 65.00	730.00 ± 170.76	656.21 ± 103.59	–
SS 2015	540.61 ± 59.47	819.28 ± 73.48	667.71 ± 73.67	760.00 ± 169.70	795.37 ± 68.75	640.00 ± 0.00
FW 2015	599.37 ± 68.78	835.63 ± 134.20	524.02 ± 32.49	–	–	–
S 2016	512.70 ± 22.14	724.38 ± 98.46	625.54 ± 27.19	596.50 ± 63.62	789.57 ± 52.57	733.63 ± 52.04

**Note:**

Average MDS calculations (±1 SE) for each species during each sampling period: spring-summer 2014 (*N* = 9 cruises), fall-winter 2014 (*N* = 6 cruises), spring-summer 2015 (*N* = 8 cruises), fall-winter 2015 (*N* = 4 cruises), summer 2016 (*N* = 6 cruises). If the average MDS value was only based on measurements from one cruise, it was excluded from this table.

In general, water temperature was relatively low at the start of spring-summer sampling periods, increased throughout spring-summer, remained high until fall, and then decreased during fall-winter sampling periods (Fig. 3A). The highest temperatures were recorded during fall-winter 2015, and the lowest measurement was during summer 2016. Thermal stratification was high during spring-summer sampling, low during fall-winter sampling, and the highest measurements occurred during the first few cruises of summer 2016 (Fig. 3A). Overall mean temperature for each sampling period ranged from 17.7 to 20.6 °C, and was higher in fall-winter than spring-summer (Fig. 5B). Mean temperature was similar during both sampling periods in 2014 and summer 2016, ranging between 18.7 and 19.0 °C, but decreased to 17.7 °C in spring-summer 2015 and increased to 20.6 °C in fall-winter 2015. Mean zonal stratification ranged from 0.05 to 0.38 °C m^−1^ and was lowest in fall-winter sampling periods (Fig. 5C). Mean zonal stratification was highest in summer 2016, and was more variable in spring-summer/summer sampling periods than fall-winter sampling periods (Figs. 3A and 5C).

## Discussion

Six species of barnacle cyprids were identified in our samples: *C. fissus*, *B. glandula*, *P. polymerus*, *T. rubescens*, *M. rosa*, and *B. trigonus*. Four cyprid morphotypes were not identified, and could possibly include *Balanoidea* sp. MM-2014, as we collected adults from that species in the intertidal habitats near our plankton sampling location. *C. fissus*, *B. glandula*, and *P. polymerus* cyprids were identified morphologically and confirmed via DNA barcoding. *T. rubescens*, *M. rosa*, and *B. trigonus* cyprids in our samples were not easily identified by morphology and were unknown until molecular analysis was completed. The results of barcoding not only confirmed our known morphological identifications, but also helped us identify some of our unknown larval morphotypes, providing confidence in our results and subsequent analyses when comparing abundances and cross-shore distributions among species. While molecular analysis added certainty to our results, we did have a low success rate overall for DNA barcoding (28%), which may be due to DNA degradation in ethanol over time. Samples were collected between May 2014 and August 2016, and barcoding was conducted in May 2016 and January 2017. The success rate increased with more recently collected specimens: 14%, 27%, and 63% success for cyprids collected in 2014, 2015, and 2016, respectively (Table 1). Additionally, it is possible that cyprids could have been contaminated with trace amounts of rose Bengal stain during the sorting process, because the same instruments were used to sort stained and unstained samples. We found that the Geller–Leray primer set had higher success than the Folmer primer set, suggesting that it is useful in barcoding and metabarcoding of barnacle larvae.

There were some similarities in timing of density peaks across species, but abundance varied both within and between sampling periods for each cyprid species. The highest recorded densities occurred at different times for different species, which could be due to variable timing of reproductive output, differences in mortality rates between species, differences in larval duration, differences in behaviors, and larval dispersal and transport. While larval distributions are known to be variable (Haury, McGowan & Wiebe, 1978), cyprid abundance was highest for five of the six species sampled at the beginning (spring-summer 2014) or end (summer 2016) of our sampling, rather than during El Niño. El Niño conditions did not arrive at our study site until April 2015, but the warm water anomaly also known as the “Blob” began affecting southern California in mid-2014, after our spring-summer 2014 sampling period started (Leising et al., 2015; Zaba & Rudnick, 2016; Pineda, Reyns & Lentz, 2018). Densities of *C. fissus*, *T. rubescens*, *B. trigonus*, and *B. glandula* cyprids had their peaks in spring-summer 2014 and summer 2016, before and after the warm water anomaly and El Niño conditions affected the area, which suggests that these species could have been negatively affected by the environmental disturbances. These results agree with findings reported by Pineda, Reyns & Lentz (2018) of low barnacle nauplii and *C. fissus* cyprid abundances in La Jolla during the “Blob” and El Niño, as well as low barnacle settlement rates. *C. fissus* cyprids have been shown to be more constrained nearshore when stratification is high (Hagerty, Reyns & Pineda, 2018), and nearshore water column stratification and variability of the cross-shore currents, which is indicative of internal wave energy, were lower during the disturbances than after (Pineda, Reyns & Lentz, 2018). A decrease in thermal stratification and internal movements known to aid in behavioral control of horizontal distribution and transport larvae shoreward, as well as a possible decrease in reproduction and increase in larval mortality, could be related to the low abundances of *C. fissus*, *T. rubescens*, *B. trigonus*, and *B. glandula* cyprids during the disturbances (Pineda, Reyns & Lentz, 2018). Fraser & Chan (2019) found that adult *Fistulobalanus albicostatus* barnacles exposed to heat stress significantly reduced their mating behavior and almost never released larvae. Water temperature at our study site increased throughout spring-summer 2015 and remained high throughout fall-winter 2015 during El Nino, so a potential explanation for lower cyprid densities is reduced reproduction in response to heat stress. It is possible that *P. polymerus* barnacles are more susceptible to changes associated with oceanic disturbances, since the density of *P. polymerus* cyprids increased greatly in summer 2016, when El Niño conditions were no longer present. *M. rosa* cyprids had high densities in spring-summer 2014, spring-summer 2015, and summer 2016, which could be related to the similarities in mean temperature and higher mean zonal stratification during these periods, and to a lack of response to El Niño.

In spring-summer 2015, at the onset of El Niño in southern California, average cyprid abundance was lowest, mainly due to a decrease of the dominant *C. fissus* cyprids. The relative proportion of *C. fissus* to total cyprids was lowest during this sampling period, while it was highest in the previous sampling period, fall-winter 2014. This supports the notion that the 2015–16 warm phase of ENSO event had a negative impact on *C. fissus* larvae in particular. We anticipated changes in species composition during El Niño because such changes have occurred for barnacles and other planktonic organisms, as well as fishes, in southern California and elsewhere (Newman & McConnaughey, 1987; Arntz & Tarazona, 1990; Warwick, Clarke & Suharsono, 1990; Davis, 2000; Carballo, Olabarria & Osuna, 2002; Navarrete et al., 2002; Garcia, Vieira & Winemiller, 2003; Lea & Rosenblatt, 2000; McClatchie et al., 2016). However, our results did not indicate an increase in species richness or a major change in the species assemblage. Relative proportions of cyprid species varied across sampling periods and cruises, but it is unclear if El Niño caused the variability. We did find that all six known cyprid species and unknown cyprids were present during all spring-summer and summer sampling periods. Unknown cyprids were not collected during fall-winter 2014 or 2015; *B. trigonus* cyprids were not present in samples from fall-winter 2014, and *T. rubescens* cyprids were not found in fall-winter 2015. Zonal stratification was lowest during fall-winter sampling periods, so it is possible that zonal stratification affects the cross-shore distribution of those species in a similar way as *C. fissus*. *C. fissus* cyprids were distributed farther offshore at our study site during fall-winter compared to spring-summer when waters were more stratified (Hagerty, Reyns & Pineda, 2018). As opposed to the 1997–98 El Niño, when anomalous poleward flow was observed in coastal Californian waters (Lynn & Bograd, 2002; Pérez-Brunius, López & Pineda, 2006), currently there is no evidence for northward flow associated with the 2015–16 El Niño (McClatchie et al., 2016), and we speculate that this might partially explain the relative constancy of the barnacle larval assemblage and why *E*(*S*_20_) did not increase during El Niño. The emergence of the “Blob,” prior to the 2015 El Niño (Leising et al., 2015; Zaba & Rudnick, 2016), may have helped obscure a clear difference in species assemblages during El Niño. In fact, at the conclusion of El Niño in summer 2016, mean temperature cooled, zonal stratification increased, and *E*(S_20_) was less variable.

Cross-shore distributions of cyprids appear to be species-specific. Across our five sampling stations located within one km from shore, there were significant differences in MDS values between species. The largest in size of the known cyprid species, *M. rosa*, was distributed significantly farther from shore than *C. fissus* and *P. polymerus*, the two smallest cyprids. Cyprids are negatively buoyant (DiBacco et al., 2011), and *M. rosa* cyprids could be located deeper in the water column than the other two species. This could cause a difference in their cross-shore distributions, as vertical distribution can control horizontal transport through exposure to varying currents (Pineda & Reyns, 2018). Further, adult *C. fissus* barnacles dominate the intertidal habitat near our sampling site, and *P. polymerus* adults are much more abundant than *M. rosa*. We only collected two adult *M. rosa* specimens, found in the intertidal at Dike Rock, La Jolla, which is slightly north of the plankton sampling location. Cross-shore larval distributions may be influenced by adult habitat locations. Average MDS for *C. fissus* was lowest in summer 2016, when zonal stratification was highest. This means that *C. fissus* cyprids were closest to shore when waters were the most stratified, which further supports both the hypothesis that cyprids have more behavioral control to accumulate nearshore in stratified waters and the previous finding of more constrained nearshore distributions of *C. fissus* cyprids when thermal stratification is high (Hagerty, Reyns & Pineda, 2018).

## Conclusions

In this study, we hypothesized that species composition of barnacle cyprids in nearshore waters off La Jolla, southern California would change during the 2015–16 ENSO disturbance. Specifically, we predicted that species diversity would increase. Our findings did not support this hypothesis, as there was no significant difference in *E*(*S*_20_), an estimation of diversity, across sampling periods. Relative species proportions and diversity varied slightly throughout the study, but there was not an increase in species richness associated with the arrival of the warm phase of El Niño. Mean *E*(*S*_20_) was slightly lower in fall-winter 2014 during the warm water anomaly and before El Niño, but it was comparable across all other sampling periods. We also wanted to determine if there were any changes in cyprid abundance caused by the warm water anomaly and El Niño, and found that cyprid abundance decreased during the environmental disturbances. Densities increased to pre-disturbance numbers or higher after El Niño conditions disappeared from the area.

We also investigated how horizontal distributions of cyprids varied by species. There were significant differences in MDS values between species, and distance from shore fluctuated by season (spring-summer vs. fall-winter) for some species. Our results reinforce previous findings that *C. fissus* cyprids are distributed closer to shore when the water column is thermally stratified. It is unclear how MDS estimates for cyprid species other than *C. fissus* reflect actual patterns, because only *C. fissus* cyprids were represented in high numbers. Additional sampling in the area could help to better define cross-shore distributions of all cyprid species. Further, supplemental sampling could result in the collection of more of the unknown morphotypes, which would allow for additional attempts at DNA barcoding and positive identifications to species.

## Supplemental Information

10.7717/peerj.7186/supp-1 Supplemental Information 1 Nucleotide sequences generated in this study.

10.7717/peerj.7186/supp-2 Supplemental Information 2 Cyprid densities and CTD cast depth and temperature readings for each cruise.

10.7717/peerj.7186/supp-3 Supplemental Information 3 Unknown adult barnacles. Photos of 2 of the unknown *Balanoidea sp*. adult barnacles collected in the intertidal at Dike Rock, La Jolla, California, USA and Alisitos, Baja California, Mexico.

## Additional Information and Declarations

### Field Study Permissions

The following information was supplied relating to field study approvals (i.e., approving body and any reference numbers): Field experiments were approved by the California Department of Fish and Wildlife scientific collection permit SC-10523.

### DNA Deposition

The following information was supplied regarding the deposition of DNA sequences: The mitochondrial COI sequences are accessible via GenBank: MK496547 to MK496616.

### Data Availability

The following information was supplied regarding data availability: The raw data are available in Supplemental File 2. Raw measurements include cyprid densities and depth and temperature readings from CTD casts.
