# Supplementary material for: Diversity and distribution of nearshore barnacle cyprids in southern California through the 2015–16 El Niño
Source: PeerJ. 2019 Jul 1;7:e7186. doi: 10.7717/peerj.7186 (PMC6610546; doi:10.7717/peerj.7186)
Supplement: Supplemental Information 1 [file peerj-07-7186-s001.docx]

>SDc1 [organism=Chthamalus fissus] [lat-lon=32.8100,-117.2738] [collection-date=23-Jun-2015] [mol_type=genomic DNA] [country=USA: North Pacific Ocean, California] [db_xref=taxon:116177]

AACATTGCTCATTCCGGAGCTTCTGTTGACTTATCTATCTTTTCTTTACATTTGGCAGGGGCTTCTTCCATTTTAGGGGCTATTAATTTTATATCTACTGTCATTAATATGCGAGCAGAGACATTAACATTTGACCGCATCCCTTTATTTGTTTGAAGTGTATTTGTGACAGTAATTTTGTTGTTGTTGTCTTTACCAGTACTAGCAGGAGCTATTACTATGCTTCTAACAGATCGTAATTTGAATACTTCATTTTTTGACCCTACCGGTGGAGGTGACCCAATCCTTTACCAACATTTATTTTGATTCTTCGGCCATCGAAGTCTAA

>SDc2 [organism=Chthamalus fissus] [lat-lon=32.8100,-117.2738] [collection-date=23-Jun-2015] [mol_type=genomic DNA] [country=USA: North Pacific Ocean, California] [db_xref=taxon:116177]

TTATCAAGTAACATTGCTCATTCCGGAGCTTCTGTTGATTTATCTATTTTTTCATTACATTTGGCAGGAGCTTCTTCCATTTTAGGGGCTATTAATTTTATATCTACTGTCATTAATATACGAGCAGAAACATTAACATTCGACCGCATCCCTTTATTTGTTTGAAGTGTATTTGTGACAGTAATTTTGTTGCTGTTATCTTTACCAGTACTAGCAGGAGCTATTACTATGCTTCTAACAGATCGTAATTTGAATACTTCATTTTTTGACCCTACCGGTGGAGGTGACCCAATCCTTTACCAACATCTATTTT

>SDc3 [organism=Chthamalus fissus] [lat-lon=32.8100,-117.2738] [collection-date=23-Jun-2015] [mol_type=genomic DNA] [country=USA: North Pacific Ocean, California] [db_xref=taxon:116177]

TTTATCAAGTAACATTGCTCATTCCGGAGCTTCTGTTGACTTATCTATTTTTTCCTTACATTTGGCAGGGGCTTCTTCCATTTTAGGGGCTATTAATTTTATATCTACTGTCATTAATATACGAGCAGAAACATTAACATTTGACCGCATCCCTTTATTTGTTTGAAGTGTATTTGTGACAGTAATTTTGTTGCTGTTATCTTTACCAGTACTAGCAGGAGCTATTACTATGCTTCTAACAGATCGTAATTTGAATACTTCATTTTTTGACCCTACTGGTGGAGGTGACCCAATCCTTTACCAACATCTATTTTG

>SDc4 [organism=Chthamalus fissus] [lat-lon=32.8100,-117.2738] [collection-date=11-Jun-2015] [mol_type=genomic DNA] [country=USA: North Pacific Ocean, California] [db_xref=taxon:116177]

GATCGGCAATGGTCGGTACAGCTTTAAGCTTACTTATTCGGGCAGAATTAGGTCAACCAGGAAGTTTAATCGGGGACGACCAGATTTACAATGTAATTGTTACAGCCCATGCTTTTATTATAATTTTCTTCATAGTTATACCTATTATAATTGGAGGTTTTGGTAATTGACTATTACCACTAATGCTTGGAGCTCCTGATATAGCTTTTCCTCGTTTAAACAATATAAGTTTTTGACTATTGCCCCCAGCACTTATACTTCTAATTAGAGGCTCATTGGTTGAAGCAGGAGCAGGAACGGGTTGAACAGTTTATCCCCCTTTATCAAGTAACATTGCTCATTCCGGAGCTTCTGTTGACTTATCTATTTTTTCCTTACATTTGGCAGGAGCTTCTTCCATTTTAGGGGCTATTAATTTTATATCTACTGTCATTAATATACGAGCAGAAACATTAACATTTGACCGCATCCCTTTATTTGTTTGAAGTGTATTTGTGACAGTAATTTTGTTGCTGTTATCTTTACCAGTACTAGCAGGAGCTATTACTATGCTTCTAACAGATCGTAATTTGAATACTTCA

>SDc5 [organism=Chthamalus fissus] [lat-lon=32.8100,-117.2738] [collection-date=22-Apr-2016] [mol_type=genomic DNA] [country=USA: North Pacific Ocean, California] [db_xref=taxon:116177]

CAAGTAACATTGCTCATTCCGGAGCTTCTGTTGACTTATCTATTTTTTCATTACATTTGGCAGGGGCTTCTTCCATTTTAGGGGCTATTAATTTTATATCTACTGTCATTAATATACGAGCAGAAACATTAACATTTGACCGCATCCCTTTATTTGTTTGAAGTGTATTTGTGACAGTAATTTTGTTGCTGTTATCTTTACCAGTACTAGCAGGAGCTATTACTATGCTTCTAACAGATCGTAATTTGAATACTTCATTTTTTGACCCTACCGGTGGAGGTGACCCAATCCTTTACCAACATCTATTTTGATT

>SDc6 [organism=Chthamalus fissus] [lat-lon=32.8100,-117.2738] [collection-date=20-Jul-2016] [mol_type=genomic DNA] [country=USA: North Pacific Ocean, California] [db_xref=taxon:116177]

TTTATCAAGTAACATTGCTCATTCCGGAGCTTCTGTTGACTTATCTATTTTTTCCTTACATTTGGCAGGGGCTTCTTCCATTTTAGGGGCTATTAATTTTATATCTACTGTCATTAATATACGAGCAGAAACATTAACATTTGACCGCATCCCTTTATTTGTTTGAAGTGTATTTGTGACAGTAATTTTGTTGCTGTTATCTTTACCAGTACTAGCAGGAGCTATTACTATGCTTCTAACAGATCGTAATTTGAATACTTCATTTTTTGACCCTACCGGTGGAGGTGACCCAATCCTTTACCAACATCTATTT

>SDc7 [organism=Chthamalus fissus] [lat-lon=32.8100,-117.2738] [collection-date=20-Jul-2016] [mol_type=genomic DNA] [country=USA: North Pacific Ocean, California] [db_xref=taxon:116177]

TTATCAAGTAACATTGCTCATTCCGGAGCTTCTGTTGACTTATCTATTTTTTCCTTACATTTGGCAGGGGCTTCTTCCATTTTAGGGGCTATTAATTTTATATCTACTGTCATTAATATACGAGCAGAAACATTAACATTTGACCGCATCCCTTTATTTGTTTGAAGTGTATTTGTGACAGTAATTTTGTTGCTGTTATCTTTACCAGTACTAGCAGGAGCTATTACTATGCTTCTAACAGATCGTAATTTGAATACTTCATTTTTTGATCCTACCGGTGGAGGTGACCCAATCCTTTACCAACATCTATTT

>SDc8 [organism=Chthamalus fissus] [lat-lon=32.8100,-117.2738] [collection-date=15-Jul-2016] [mol_type=genomic DNA] [country=USA: North Pacific Ocean, California] [db_xref=taxon:116177]

TCAAGTAACATTGCTCATTCCGGAGCTTCTGTTGACTTATCTATTTTTTCCTTACATTTGGCAGGGGCTTCTTCCATTTTAGGGGCTATTAATTTTATATCTACTGTCATTAATATACGAGCAGAAACATTAACATTTGACCGCATCCCTTTATTTGTTTGAAGTGTATTTGTGACAGTAATTTTGTTGCTGTTATCTTTACCGGTACTAGCAGGAGCTATTACTATGCTTTTAACAGATCGTAATTTGAATACTTCATTTTTTGACCCTACCGGTGGAGGTGACCCAATCCTTTATCAACATCTATTTT

>SDc9 [organism=Chthamalus fissus] [lat-lon=32.8100,-117.2738] [collection-date=15-Jul-2016] [mol_type=genomic DNA] [country=USA: North Pacific Ocean, California] [db_xref=taxon:116177]

TTATCAAGTAACATTGCTCATTCCGGAGCTTCTGTTGACTTATCTATTTTTTCCTTACATTTGGCAGGGGCTTCTTCCATTTTAGGAGCTATTAATTTTATATCTACTGTCATTAATATACGAGCAGAAACATTAACATTTGACCGCATCCCTTTATTTGTTTGAAGTGTATTTGTGACAGTAATTTTGTTGCTGTTATCTTTACCAGTACTAGCAGGAGCTATTACTATGCTTCTAACAGATCGTAATTTGAATACTTCATTTTTTGACCCTACCGGTGGAGGTGACCCAATCCTTTACCAACATCTATTT

>SDc10 [organism=Chthamalus fissus] [lat-lon=32.8100,-117.2738] [collection-date=15-Jul-2016] [mol_type=genomic DNA] [country=USA: North Pacific Ocean, California] [db_xref=taxon:116177]

TATCAAGTAACATTGCTCATTCCGGAGCTTCTGTTGACTTATCTATTTTTTCCTTACATTTGGCAGGGGCTTCTTCCATTTTAGGGGCTATTAATTTTATATCTACTGTCATTAATATACGAGCAGAAACATTAACATTTGACCGTATCCCTTTATTTGTTTGAAGTGTATTTGTGACAGTAATTTTGTTGCTGTTATCTTTACCAGTACTAGCAGGAGCTATTACTATGCTTCTAACAGATCGTAATTTGAATACTTCATTTTTTGACCCTACCGGTGGAGGTGACCCAATCCTTTACCAACATCTATTT

>SDc11 [organism=Chthamalus fissus] [lat-lon=32.8100,-117.2738] [collection-date=06-Nov-2015] [mol_type=genomic DNA] [country=USA: North Pacific Ocean, California] [db_xref=taxon:116177]

TTATCAAGTAACATTGCTCATTCCGGAGCTTCTGTTGACTTATCTATTTTTTCATTACATTTGGCAGGGGCTTCTTCCATTTTAGGGGCTATTAATTTTATATCTACTGTCATTAATATACGAGCAGAAACATTAACATTTGACCGCATCCCTTTATTTGTTTGAAGTGTATTTGTGACAGTAATTTTGTTGCTGTTATCTTTACCAGTACTAGCAGGAGCTATTACTATGCTTTTAACAGATCGTAATTTGAATACTTCATTTTTTGACCCTACCGGTGGAGGTGACCCAATCCTTTACCAACATCTATTC

>SDc12 [organism=Balanus glandula] [lat-lon=32.8100,-117.2738] [collection-date=23-May-2014] [mol_type=genomic DNA] [country=USA: North Pacific Ocean, California] [db_xref=taxon:110520]

GATTGGAGATGATCAGATTTACAATGTAATTGTTACTGCTCATGCTTTTATTATGATTTTTTTCATAGTTATACCTATTATAATTGGGGGTTTTGGTAATTGATTACTTCCATTAATATTAGGAGCTCCTGATATAGCTTTTCCACGTCTTAATAATATAAGTTTTTGGCTTTTACCCCCAGCTTTAATATTGTTGATTAGAGGATCATTAGTAGAAGCTGGAGCTGGTACTGGGTGGACAGTTTACCCTCCTTTATCGAGAAATATCGCCCATTCAGGAGCATCGGTAGATTTATCTATTTTTTCTCTCCATTTAGCTGGAGCTTCATCTATTCTTGGGGCCATTAATTTTATATCAACAGTTATTAATATGCGAGCAGAGACTTTAACGTTTGATCGTCTTCCTTTATTTGTGTGAAGTGTTTTTATTACTGTAATCTTACTTTTACTGTCTCTACCTGTATTAGC

>SDc13 [organism=Balanus glandula] [lat-lon=32.8089,-117.2795] [collection-date=28-Apr-2015] [mol_type=genomic DNA] [country=USA: North Pacific Ocean, California] [db_xref=taxon:110520]

ATCGAGAAATATCGCCCATTCAGGAGCATCGGTAGATTTATCTATTTTTTCTCTCCATTTAGCTGGAGCTTCATCTATTCTTGGGGCCATTAATTTTATATCAACAGTTATTAATATGCGAGCAGAGACTTTAACGTTTGATCGTCTTCCTTTATTTGTGTGAAGTGTTTTTATTACTGTAATCTTACTTTTACTATCTCTACCTGTATTAGCTGGAGCTATTACAATATTACTAACAGATCGAAATTTGAATACATCATTTTTTGACCCAACTGGAGGAGGTGATCCGATTTTATACCAACACTTATTC

>SDc14 [organism=Balanus glandula] [lat-lon=32.8073,-117.2794] [collection-date=28-Apr-2015] [mol_type=genomic DNA] [country=USA: North Pacific Ocean, California] [db_xref=taxon:110520]

TTATCTATTTTTTCTCTCCATTTAGCTGGAGCTTCATCTATTCTTGGGGCCATTAATTTTATATCAACAGTTATTAATATGCGAGCAGAGACTTTAACGTTTGATCGTCTTCCTTTATTTGTGTGAAGTGTTTTTATTACTGTAATCTTACTTTTACTGTCTCTACCTGTATTAGCTGGAGCTATTACAATATTACTAACAGATCGAAATTTAAATACATCATTTTTTGACCCAACTGGAGGAGGTGATCCGATTTTATACCAACACTTATTC

>SDc15 [organism=Pollicipes polymerus] [lat-lon=32.8100,-117.2738] [collection-date=18-Jun-2016] [mol_type=genomic DNA] [country=USA: North Pacific Ocean, California] [db_xref=taxon: 36137]

CCAGCAATATCGCACACTCAGGAGCCTCTGTAGACCTCTCTATTTTTTCATTACACTTAGCGGGAGCTTCCTCTATTTTAGGAGCTATCAACTTCATATCCACAGTAATTAATATACGAGCTGAAACTTTAACATTCGACCGTTTACCTTTATTTGTATGAAGAGTATTTGTGACGGTGATTCTACTATTATTATCTTTACCTGTGTTAGCCGGAGCCATTACTATGCTTTTAACTGACCGGAATCTTAATACATCATTTTTCGACCCTACAGGGGGAGGAGACCCCATTTTATACCAACACTTATT

>SDc16 [organism=Tetraclita rubescens] [lat-lon=32.8089,-117.2795] [collection-date=28-Apr-2015] [mol_type=genomic DNA] [country=USA: North Pacific Ocean, California] [db_xref=taxon: 311010]

AGTAATATTGCCCACTCAGGAGCCTCAGTAGATTTATCTATTTTTTCCTTGCATTTAGCAGGAGCTTCTTCCATTCTAGGTGCTATTAATTTTATATCCACAGTTATTAATATACGAGCAGAAACTTTAACTTTCGATCGTCTTCCTCTTTTTGTATGAAGTGTTTTTATTACTGTAATTCTTCTTCTTTTATCTCTCCCAGTTTTAGCAGGGGCTATTACTATACTTCTAACCGATCGAAATCTTAATACATCTTTTTTTGATCCAACTGGAGGAGGAGACCCTATTCTGTATCAACATTTATTC

>SDc17 [organism=Tetraclita rubescens] [lat-lon=32.8089,-117.2795] [collection-date=28-Apr-2015] [mol_type=genomic DNA] [country=USA: North Pacific Ocean, California] [db_xref=taxon: 311010]

GCTTCTTCCATTCTAGGTGCTATTAATTTTATATCCACAGTTATTAATATACGAGCAGAAACTTTAACTTTCGATCGTCTTCCTCTTTTTGTATGAAGTGTTTTTATTACTGTAATTCTTCTTCTTTTATCTCTCCCAGTTTTAGCAGGGGCTATTACTATACTTCTAACCGATCGAAATCTTAATACATCTTTTTTTGATCCAACTGGAGGAGGAGACCCTATTCTGTATCAACATT

>SDc18 [organism=Tetraclita rubescens] [lat-lon=32.8100,-117.2738] [collection-date=22-Jul-2015] [mol_type=genomic DNA] [country=USA: North Pacific Ocean, California] [db_xref=taxon: 311010]

AGTAATATTGCCCACTCAGGAGCCTCAGTAGACTTATCTATTTTTTCCTTGCATTTAGCAGGAGCTTCTTCCATTCTAGGTGCTATTAATTTTATATCCACAGTTATTAATATACGAGCAGAAACTTTAACTTTCGATCGTCTTCCTCTTTTTGTATGAAGTGTTTTTATTACTGTAATTCTTCTTCTTTTATCTCTCCCAGTTTTAGCAGGGGCTATTACTATACTTCTAACCGATCGAAATCTTAATACATCTTTTTTTGATCCAACTGGAGGAGGAGACCCTATTCTGTATCAACATTTATTC

>SDc19 [organism=Tetraclita rubescens] [lat-lon=32.8100,-117.2738] [collection-date=23-Jul-2015] [mol_type=genomic DNA] [country=USA: North Pacific Ocean, California] [db_xref=taxon: 311010]

TAGTAATATTGCCCACTCAGGAGCCTCAGTAGATTTATCTATTTTTTCCTTGCATTTAGCAGGAGCTTCTTCCATTCTAGGTGCTATTAATTTTATATCCACAGTTATTAATATACGAGCAGAAACTTTAACTTTCGATCGTCTTCCTCTTTTTGTATGAAGTGTTTTTATTACTGTAATTCTTCTTCTTTTATCTCTCCCAGTTTTAGCAGGGGCTATTACTATACTTCTAACCGATCGAAATCTTAATACATCTTTTTTTGATCCAACTGGAGGAGGAGACCCTATTCTGTATCAACATTTATT

>SDc20 [organism=Tetraclita rubescens] [lat-lon=32.8100,-117.2738] [collection-date=23-Jul-2015] [mol_type=genomic DNA] [country=USA: North Pacific Ocean, California] [db_xref=taxon: 311010]

CTAGTAATATTGCCCACTCAGGAGCCTCAGTAGATTTATCTATTTTTTCCTTGCATTTAGCAGGAGCTTCTTCCATTCTAGGTGCTATTAATTTTATATCCACAGTTATTAATATACGAGCAGAAACTTTAACTTTCGATCGTCTTCCTCTTTTTGTATGAAGTGTTTTTATTACTGTAATTCTTCTTCTTTTATCTCTCCCAGTTTTAGCAGGGGCTATTACTATACTTCTAACCGATCGAAATCTTAATACATCTTTTTTTGATCCAACTGGAGGAGGAGACCCTATTCTGTATCAACATTTATT

>SDc21 [organism=Tetraclita rubescens] [lat-lon=32.8073,-117.2794] [collection-date=09-May-2014] [mol_type=genomic DNA] [country=USA: North Pacific Ocean, California] [db_xref=taxon: 311010]

TCAGGAGCCTCAGTAGATTTATCTATTTTTTCCTTGCATTTAGCAGGAGCTTCTTCCATTCTAGGTGCTATTAATTTTATATCCACAGTTATTAATATACGAGCAGAAACTTTAACTTTCGATCGTCTTCCTCTTTTTGTATGAAGTGTTTTTATTACTGTAATTCTTCTTCTTTTATCTCTCCCAGTTTTAGCAGGGGCTATTACTATACTTCTAACTGATCGAAATCTTAATACATCTTTTTTTGATCCAACTGGAGGAGGAGACCCTATTCTGTATCAACATTTATTC

>SDc22 [organism=Megabalanus rosa] [lat-lon=32.8089,-117.2795] [collection-date=23-May-2014] [mol_type=genomic DNA] [country=USA: North Pacific Ocean, California] [db_xref=taxon: 6680]

CAAGTAATATTGCTCACTCAGGTGCTTCTGTAGATTTATCTATTTTTTCTTTACACTTAGCTGGAGCATCCTCAATTCTAGGTGCAATTAATTTTATGTCTACAGTTATTAACATACGAGCAGAAACTTTAACATTTGACCGTCTACCTTTGTTCGTGTGAAGAGTTTTCATTACTGTTATTTTACTATTACTTTCCTTACCAGTTTTAGCAGGTGCAATTACTATGTTGTTAACTGACCGTAATCTTAACACCTCATTCTTTGACCCTACAGGTGGAGGAGACCCTATTCTTTACCAACACTTAT

>SDc23 [organism=Megabalanus rosa] [lat-lon=32.8073,-117.2794] [collection-date=28-Apr-2015] [mol_type=genomic DNA] [country=USA: North Pacific Ocean, California] [db_xref=taxon: 6680]

TTATCAAGTAATATTGCTCACTCAGGTGCTTCTGTAGATTTATCTATTTTTTCTTTACACTTAGCTGGAGCATCCTCAATTCTAGGTGCAATTAATTTTATGTCTACAGTTATTAATATACGAGCAGAAACTTTAACATTTGATCGCCTACCTTTGTTCGTGTGAAGAGTTTTCATTACTGTTATTTTACTATTACTTTCCTTACCAGTTTTAGCAGGTGCAATTACTATGTTGTTAACTGACCGTAATCTTAACACCTCATTCTTTGATCCTACAGGTGGAGGAGACCCTATTCTTTACCAACACTTATTT

>SDc24 [organism=Megabalanus rosa] [lat-lon=32.8073,-117.2794] [collection-date=28-Apr-2015] [mol_type=genomic DNA] [country=USA: North Pacific Ocean, California] [db_xref=taxon: 6680]

TATCAAGTAATATTGCTCACTCAGGTGCTTCTGTAGATTTATCTATTTTTTCTTTACACTTAGCTGGGGCATCCTCAATTCTAGGTGCAATTAATTTTATGTCTACAGTTATTAATATACGAGCAGAAACTTTAACATTTGACCGTTTACCTTTGTTCGTGTGAAGAGTTTTCATTACTGTTATTTTACTATTACTTTCCTTACCAGTTTTAGCAGGTGCAATTACTATGTTGTTAACTGACCGTAATCTTAACACCTCATTCTTTGACCCTACAGGTGGAGGAGACCCTATTCTTTACCAACACTTATTT

>SDc25 [organism=Megabalanus rosa] [lat-lon=32.8073,-117.2794] [collection-date=09-May-2014] [mol_type=genomic DNA] [country=USA: North Pacific Ocean, California] [db_xref=taxon: 6680]

TTTATCAAGTAATATTGCTCATTCAGGTGCTTCTGTAGATTTATCTATTTTTTCTTTACACTTAGCTGGAGCATCCTCAATTCTAGGTGCAATTAATTTTATGTCTACAGTTATTAATATACGAGCAGAAACTTTAACATTTGACCGTCTACCTTTGTTCGTGTGAAGAGTTTTCATTACTGTTATTTTACTATTACTTTCCTTACCAGTTTTAGCAGGTGCAATTACTATGTTGTTAACTGACCGTAATCTTAACACCTCATTCTTTGACCCTACAGGTGGAGGAGACCCTATTCTTTACCAACACTTATTTT

>SDc26 [organism=Balanus trigonus] [lat-lon=32.8073,-117.2794] [collection-date=16-Oct-2015] [mol_type=genomic DNA] [country=USA: North Pacific Ocean, California] [db_xref=taxon: 756461]

AGCTTCCGTAGATTTATCGATTTTTTCTTTACACTTAGCGGGAGCTTCATCAATTTTAGGAGCTATTAATTTTATATCGACTGTTATTAATATGCGAGCTGAAACATTAACATTTGACCGTCTTCCATTATTTGTGTGAAGAGTATTTATTACTGTAATTCTTTTATTACTTTCACTACCAGTATTAGCTGGAGCAATTACAATGTTATTAACTGACCGAAACCTAAATACTTCATTTTTTGACCCAACTGGAGGAGGTGACCCTATTTTATACCAAC

>SDa1 [organism=Chthamalus fissus] [lat-lon=32.8098,-117.2693] [collection-date=2016-05-07] [mol_type=genomic DNA] [country=USA: North Pacific Ocean, California] [db_xref=taxon:116177]

ATCAAGTAACATTGCTCATTCCGGAGCTTCTGTTGACTTATCTATTTTTTCCCTACATTTGGCAGGGGCTTCTTCCATTTTAGGGGCTATTAATTTTATATCTACTGTCATTAATATACGAGCAGAAACATTAACATTTGACCGCATCCCTTTATTTGTTTGAAGTGTATTTGTGACAGTAATTTTGTTGCTGTTATCTTTACCAGTACTAGCAGGAGCTATTACTATGCTTCTAACAGATCGTAATTTGAATACTTCATTTTTTGATCCTACCGGTGGAGGTGACCCAATCCTTTACCAACATCTATTT

>SDa2 [organism=Chthamalus fissus] [lat-lon=32.8098,-117.2693] [collection-date=2016-05-07] [mol_type=genomic DNA] [country=USA: North Pacific Ocean, California] [db_xref=taxon:116177]

CAAGTAACATTGCTCATTCCGGAGCTTCTGTTGACTTATCTATTTTTTCCTTACATTTGGCAGGGGCTTCTTCCATTTTAGGGGCTATTAATTTTATATCTACTGTCATTAATATACGAGCAGAAACATTAACATTTGACCGCATCCCCTTATTTGTTTGAAGTGTATTTGTGACAGTAATTTTGTTGCTGTTATCTTTACCAGTACTAGCAGGAGCTATTACTATGCTTCTAACAGATCGTAATTTGAATACTTCATTTTTTGACCCTACTGGTGGAGGTGACCCAATCCTTTATCAACATCTATTT

>SDa3 [organism=Chthamalus fissus] [lat-lon=32.8098,-117.2693] [collection-date=2016-05-07] [mol_type=genomic DNA] [country=USA: North Pacific Ocean, California] [db_xref=taxon:116177]

CAAGTAATATTGCTCATTCCGGAGCTTCTGTTGACTTATCTATTTTTTCATTACACTTGGCAGGGGCTTCTTCCATTTTAGGGGCTATTAATTTTATATCTACTGTCATTAATATACGAGCAGAAACATTAACATTTGACCGTATCCCTTTATTTGTTTGAAGTGTATTTGTGACAGTAATTCTGTTGCTGTTATCTTTACCAGTACTAGCAGGAGCTATTACTATGCTTCTAACAGATCGTAATTTGAATACTTCATTTTTTGACCCTACCGGTGGAGGTGACCCAATCCTTTACCAACATTTATTTTG

>SDa4 [organism=Chthamalus fissus] [lat-lon=32.8098,-117.2693] [collection-date=2016-05-07] [mol_type=genomic DNA] [country=USA: North Pacific Ocean, California] [db_xref=taxon:116177]

CAAGTAACATTGCTCATTCCGGAGCTTCTGTTGACTTATCTATTTTTTCATTACATTTGGCAGGGGCTTCTTCTATTTTAGGGGCTATTAATTTTATATCTACTGTCATTAATATACGAGCAGAAACATTAACATTTGACCGCATCCCTTTATTTGTTTGAAGTGTATTTGTGACAGTAATTTTGTTGCTGTTATCTTTACCAGTACTAGCAGGAGCTATTACTATGCTTCTAACAGATCGTAATTTGAATACTTCATTTTTTGACCCTACCGGTGGAGGTGACCCAATCCTTTACCAACATCTATTT

>SDa5 [organism=Chthamalus fissus] [lat-lon=32.1331,-116.8882] [collection-date=2016-06-22] [mol_type=genomic DNA] [country=USA: North Pacific Ocean, California] [db_xref=taxon: 116177]

TTTATCAAGTAACATTGCTCATTCCGGAGCTTCTGTTGACTTATCTATTTTTTCATTACATTTGGCAGGGGCTTCTTCCATTTTAGGGGCTATTAATTTTATATCTACTGTCATTAATATACGAGCAGAAACATTAACATTTGACCGCATCCCTTTATTTGTTTGAAGTGTATTTGTGACAGTAATTTTGTTGCTGTTATCTTTACCAGTACTAGCAGGAGCTATTACTATGCTTCTAACAGATCGTAATTTGAATACTTCATTTTTTGACCCTACCGGTGGAGGTGACCCAATCCTTTACCAACATCTATTTTG

>SDa6 [organism=Chthamalus fissus] [lat-lon=32.1331,-116.8882] [collection-date=2016-06-22] [mol_type=genomic DNA] [country=USA: North Pacific Ocean, California] [db_xref=taxon: 116177]

TTTATCAAGTAACATTGCTCATTCCGGAGCTTCTGTTGACTTATCTATTTTTTCCTTACATTTGGCAGGGGCTTCTTCCATTTTAGGGGCTATTAATTTTATATCTACTGTCATTAATATACGAGCAGAAACATTAACATTTGACCGCATCCCTTTATTTGTTTGAAGCGTATTTGTGACAGTAATTTTGTTGCTGTTATCTTTACCAGTACTAGCAGGAGCTATTACTATGCTTCTAACAGATCGTAATTTGAATACTTCATTTTTTGACCCTACCGGTGGAGGTGACCCAATCCTTTACCAACATCTATTTTG

>SDa7 [organism=Chthamalus fissus] [lat-lon=32.1331,-116.8882] [collection-date=2016-06-22] [mol_type=genomic DNA] [country=USA: North Pacific Ocean, California] [db_xref=taxon: 116177]

CCTTTATCAAGTAACATTGCTCATTCCGGAGCTTCTGTTGACTTATCTATTTTTTCCTTACATTTGGCAGGGGCTTCTTCCATTTTAGGGGCTATTAATTTTATATCTACTGTCATTAATATACGAGCAGAAACATTAACATTTGACCGCATCCCTTTATTTGTTTGAAGTGTATTTGTGACAGTAATTTTGTTGCTGTTATCTTTACCAGTACTAGCAGGAGCTATTACTATGCTTCTAACAGATCGTAATTTGAATACTTCATTTTTTGACCCTACCGGTGGAGGTGACCCAATCCTTTACCAACATCTATTTTG

>SDa8 [organism=Chthamalus fissus] [lat-lon=32.1331,-116.8882] [collection-date=2016-06-22] [mol_type=genomic DNA] [country=USA: North Pacific Ocean, California] [db_xref=taxon: 116177]

CCCTTTATCAAGTAACATTGCTCATTCCGGAGCTTCTGTTGACTTATCTATTTTTTCATTACATTTGGCAGGGGCTTCTTCCATTTTAGGGGCTATTAATTTTATATCTACTGTCATTAATATACGAGCAGAAACATTAACATTTGACCGCATCCCTTTATTTGTTTGAAGTGTATTTGTGACAGTAATTTTGTTGCTGTTATCTTTACCAGTACTAGCAGGAGCTATTACTATGCTTCTTACAGATCGTAATTTGAATACTTCATTTTTTGACCCTACCGGTGGAGGTGACCCAATCCTTTACCAACATCTATTTT

>SDa9 [organism=Chthamalus fissus] [lat-lon=32.1331,-116.8882] [collection-date=2016-06-22] [mol_type=genomic DNA] [country=USA: North Pacific Ocean, California] [db_xref=taxon: 116177]

TTCTGTTGACTTATCTATTTTTTCCTTACATTTGGCAGGGGCTTCTTCCATTTTAGGGGCTATTAATTTTATATCTACTGTCATTAATATACGAGCAGAAACATTAACATTTGACCGCATCCCTTTATTTGTTTGAAGCGTATTTGTGACAGTAATTTTGTTGCTGTTATCTTTACCAGTACTAGCAGGAGCTATTACTATGCTTCTAACAGATCGTAATTTGAATACTTCATTTTTTGACCCTACCGGTGGA

>SDa10 [organism=Balanus glandula] [lat-lon=32.8694,-117.2535] [collection-date=2016-06-20] [mol_type=genomic DNA] [country=USA: North Pacific Ocean, California] [db_xref=taxon:110520]

TTTATCGAGAAATATCGCCCATTCAGGAGCATCGGTAGATTTATCTATTTTTTCTCTCCATTTAGCTGGAGCTTCATCTATTCTTGGGGCCATTAATTTTATATCAACAGTTATTAATATGCGAGCAGAGACTTTAACGTTTGATCGTCTCCCTTTATTTGTGTGAAGTGTTTTTATTACTGTAATCTTACTTTTACTGTCTCTACCTGTATTAGCTGGAGCTATTACAATATTACTAACAGATCGAAATTTGAATACATCATTTTTTGACCCAACTGGAGGAGGTGATCCGATTTTATACCAACACTTATTC

>SDa11 [organism=Balanus glandula] [lat-lon=32.8694,-117.2535] [collection-date=2016-06-20] [mol_type=genomic DNA] [country=USA: North Pacific Ocean, California] [db_xref=taxon:110520]

TAGATTTATCTATTTTTTCTCTTCATTTAGCTGGAGCTTCATCTATTCTTGGGGCCATTAATTTTATATCAACAGTTATTAATATGCGAGCAGAGACTTTAACGTTTGATCGTCTTCCTTTATTTGTGTGAAGTGTTTTTATTACTGTAATCTTACTTTTACTATCTCTACCTGTATTAGCTGGAGCTATTACAATATTACTAACAGATCGAAATTTGAATACATCATTTTTTGACCCAACTGGAGGAGG

>SDa12 [organism=Balanus glandula] [lat-lon=32.8694,-117.2535] [collection-date=2016-06-20] [mol_type=genomic DNA] [country=USA: North Pacific Ocean, California] [db_xref=taxon:110520]

TTTATCGAGAAATATCGCCCATTCAGGAGCATCGGTAGATTTATCTATTTTTTCTCTCCATTTAGCTGGAGCTTCATCTATTCTTGGGGCCATTAATTTTATATCAACAGTAATTAATATGCGAGCAGAGACTTTAACGTTTGATCGTCTTCCTTTATTTGTGTGAAGTGTTTTTATTACTGTAATCTTACTTTTACTATCTCTGCCTGTATTAGCTGGAGCTATTACAATATTACTAACAGATCGAAATTTGAATACATCATTTTTTGACCCAACTGGAGGAGGTGATCCGATTTTATACCAACACTTATTC

>SDa13 [organism=Balanus glandula] [lat-lon=32.8694,-117.2535] [collection-date=2016-06-20] [mol_type=genomic DNA] [country=USA: North Pacific Ocean, California] [db_xref=taxon:110520]

CAGGAGCATCGGTAGATTTATCTATTTTTTCTCTCCATTTAGCTGGAGCTTCATCTATTCTTGGGGCCATTAATTTTATATCAACAGTTATTAATATGCGAGCAGAGACTTTAACGTTTGATCGTCTTCCTTTATTTGTGTGAAGTGTTTTTATTACTGTAATCTTACTTTTACTATCTCTACCTGTATTAGCTGGAGCTATTACAATATTACTAACAGATCGAAATTTGAATACATCATTTTTTGACCCAACTGGAGGAGGTGATCCGATTTTATACCAACACTTATTC

>SDa14 [organism=Balanus glandula] [lat-lon=32.8694,-117.2535] [collection-date=2016-06-20] [mol_type=genomic DNA] [country=USA: North Pacific Ocean, California] [db_xref=taxon:110520]

TTTATCGAGAAATATCGCCCATTCAGGAGCATCGGTAGATTTATCTATTTTTTCTCTCCATTTAGCTGGAGCTTCATCTATTCTTGGGGCCATTAATTTTATATCAACAGTTATTAATATGCGAGCAGAGACTTTAACGTTTGATCGTCTTCCTTTATTTGTGTGAAGTGTTTTTATTACTGTAATCTTACTTTTACTATCTCTACCTGTATTAGCTGGAGCTATTACAATATTACTAACAGATCGAAATTTGAATACATCATTTTTTGACCCAACTGGAGGAGGTGATCCGATTTTATACCAACACTTATTC

>SDa15 [organism=Balanus glandula] [lat-lon=32.8694,-117.2535] [collection-date=2016-06-20] [mol_type=genomic DNA] [country=USA: North Pacific Ocean, California] [db_xref=taxon:110520]

ATCGAGAAATATCGCCCATTCAGGAGCATCGGTAGATTTATCTATTTTTTCTCTCCATTTAGCTGGAGCTTCATCTATTCTTGGGGCCATTAATTTTATATCAACAGTTATTAATATGCGAGCAGAGACTTTAACGTTTGATCGTCTTCCTTTATTTGTGTGAAGTGTTTTTATTACTGTAATCTTACTTTTACTGTCTCTACCTGTATTAGCTGGAGCTATTACAATATTACTAACAGATCGAAATTTGAATACATCATTTTTTGACCCAACTGGAGGAGGTGATCCGATTTTATACCAACACTTATTC

>SDa16 [organism=Balanus glandula] [lat-lon=32.8694,-117.2535] [collection-date=2016-06-20] [mol_type=genomic DNA] [country=USA: North Pacific Ocean, California] [db_xref=taxon:110520]

TTTATCGAGAAATATCGCCCATTCAGGAGCATCGGTAGATTTATCTATTTTTTCTCTCCATTTAGCTGGAGCTTCATCTATTCTTGGGGCCATTAATTTTATATCAACAGTTATTAATATGCGAGCAGAGACTTTAACGTTTGATCGTCTTCCTTTATTTGTGTGAAGTGTTTTTATTACTGTAATCTTACTTTTACTGTCTCTACCTGTATTAGCTGGAGCTATTACAATATTACTAACAGATCGAAATTTGAATACATCATTTTTTGACCCAACTGGAGGAGGTGATCCGATTTTATACCAACAC

>SDa17 [organism=Balanus glandula] [lat-lon=32.8694,-117.2535] [collection-date=2016-06-20] [mol_type=genomic DNA] [country=USA: North Pacific Ocean, California] [db_xref=taxon:110520]

TCGAGAAATATCGCCCATTCAGGAGCATCGGTAGATTTATCTATTTTTTCTCTCCATTTAGCTGGAGCTTCATCTATTCTTGGGGCCATTAATTTTATATCAACAGTTATTAATATGCGAGCAGAGACTTTAACGTTTGATCGTCTTCCTTTATTTGTGTGAAGTGTTTTTATTACTGTAATCTTACTTTTACTGTCTCTACCTGTATTAGCTGGAGCTATTACAATATTACTAACAGATCGAAATTTGAATACATCATTTTTTGACCCAACTGGAGGAGGTGATCCGATTTTATACCAACACTTATTC

>SDa18 [organism=Balanus glandula] [lat-lon=32.8694,-117.2535] [collection-date=2016-06-20] [mol_type=genomic DNA] [country=USA: North Pacific Ocean, California] [db_xref=taxon:110520]

TTATCGAGAAATATCGCCCATTCAGGAGCATCGGTAGATTTATCTATTTTTTCTCTCCATTTAGCTGGAGCTTCATCTATTCTTGGGGCCATTAATTTTATATCAACAGTAATTAATATGCGAGCAGAGACTTTAACGTTTGATCGTCTTCCTTTATTTGTGTGAAGTGTTTTTATTACTGTAATCTTACTTTTACTATCTCTACCTGTATTAGCTGGAGCTATTACAATATTACTAACAGATCGAAATTTGAATACATCATTTTTTGACCCAACTGGAGGAGGTGATCCGATTTTATACCAACACTTATTC

>SDa19 [organism=Balanus glandula] [lat-lon=32.1331,-116.8882] [collection-date=2016-06-22] [mol_type=genomic DNA] [country=USA: North Pacific Ocean, California] [db_xref=taxon:110520]

CTCTCCATTTAGCTGGAGCTTCATCTATTCTTGGGGCCATTAATTTTATATCAACAGTTATTAATATGCGAGCAGAGACTTTAACGTTTGATCGTCTTCCTTTATTTGTGTGAAGTGTTTTTATTACTGTAATCTTACTTTTACTGTCTCTACCTGTATTAGCTGGGGCTATTACAATATTACTAACAGATCGAAATTTGAATACATCA

>SDa20 [organism=Balanus glandula] [lat-lon=32.1331,-116.8882] [collection-date=2016-06-22] [mol_type=genomic DNA] [country=USA: North Pacific Ocean, California] [db_xref=taxon:110520]

CTCTCCATTTAGCTGGAGCTTCATCTATTCTTGGGGCCATTAATTTTATATCAACAGTTATTAATATACGAGCAGAGACTTTAACGTTTGATCGTCTTCCTTTATTTGTGTGAAGTGTTTTTATTACTGTAATCTTACTTTTACTGTCTCTACCTGTATTAGCTGGAGCTATTACAATATTACTAACAGATCGAAATTTAAATACATC

>SDa22 [organism=Balanus glandula] [lat-lon=32.1331,-116.8882] [collection-date=2016-06-22] [mol_type=genomic DNA] [country=USA: North Pacific Ocean, California] [db_xref=taxon:110520]

CGAGAAATATTGCCCATTCAGGAGCATCGGTAGATTTATCTATTTTTTCTCTCCATTTAGCTGGAGCTTCATCTATTCTTGGGGCCATTAATTTTATATCAACAGTTATTAATATGCGAGCAGAGACTTTAACGTTTGATCGTCTTCCTTTATTTGTGTGAAGTGTTTTTATTACTGTAATCTTACTTTTACTATCTCTACCTGTATTAGCTGGAGCTATTACAATATTACTAACAGATCGAAATTTGAATACATCATTTTTTGACCCAACTGGAGGGGGTGATCCGATTTTATACCAAC

>SDa23 [organism=Balanus glandula] [lat-lon=32.1331,-116.8882] [collection-date=2016-06-22] [mol_type=genomic DNA] [country=USA: North Pacific Ocean, California] [db_xref=taxon:110520]

CGTAGATTTATCTATTTTTTCTCTCCATTTAGCTGGAGCTTCATCTATTCTTGGGGCCATTAATTTTATATCAACAGTTATTAATATGCGAGCGGAGACTTTAACGTTTGATCGTCTTCCTTTATTTGTGTGAAGTGTTTTTATTACTGTAATCTTACTTTTACTATCTCTACCTGTATTAGCTGGAGCTATTACAATATTACTAACAGATCGAAATTTGAATACATCATTTTTTGACCCAACTGGAGGAGG

>SDa24 [organism=Balanus glandula] [lat-lon=32.1331,-116.8882] [collection-date=2016-06-22] [mol_type=genomic DNA] [country=USA: North Pacific Ocean, California] [db_xref=taxon:110520]

TCTCTCCATTTAGCTGGAGCTTCATCTATTCTTGGGGCCATTAATTTTATATCAACAGTTATTAATATGCGAGCAGAGACTTTAACATTTGATCGTCTTCCTTTATTTGTGTGAAGTGTTTTTATTACTGTAATCTTACTTTTACTATCTCTACCTGTATTAGCTGGAGCTATTACAATATTACTAACAGATCGAAATTTGAATACATC

>SDa25 [organism=Balanus glandula] [lat-lon=32.1331,-116.8882] [collection-date=2016-06-22] [mol_type=genomic DNA] [country=USA: North Pacific Ocean, California] [db_xref=taxon:110520]

TTTAGCTGGAGCTTCATCTATTCTTGGGGCCATTAATTTTATATCAACAGTTATTAATATGCGAGCAGAGACTTTAACGTTTGATCGTCTTCCTTTATTTGTGTGAAGTGTTTTTATTACTGTAATCTTACTTTTACTATCTCTACCTGTATTAGCTGGAGCTATTACAATATTACTAACAGATCGAAATTTGAATACATCA

>SDa26 [organism=Balanus glandula] [lat-lon=32.1331,-116.8882] [collection-date=2016-06-22] [mol_type=genomic DNA] [country=USA: North Pacific Ocean, California] [db_xref=taxon:110520]

TCGAGAAATATCGCCCATTCAGGAGCATCGGTAGATTTATCTATTTTTTCTCTCCATTTAGCTGGAGCTTCATCTATTCTTGGGGCCATTAATTTTATATCAACAGTTATTAATATGCGAGCAGAGACTTTAACATTTGATCGTCTTCCTTTATTTGTGTGAAGTGTTTTTATTACTGTAATCTTACTTTTACTATCTCTACCTGTATTAGCTGGAGCTATTACAATATTACTAACAGATCGAAATTTGAATACATCATTTTTTGACCCAACTGGAGGAGGTGATCCGATTTTATACCAACACTTATTCTGATTCTTCGGCCACCCGAAGTCTAA

>SDa27 [organism=Balanus glandula] [lat-lon=32.1331,-116.8882] [collection-date=2016-06-22] [mol_type=genomic DNA] [country=USA: North Pacific Ocean, California] [db_xref=taxon:110520]

CATTTAGCTGGAGCTTCATCTATTCTTGGGGCCATTAATTTTATATCAACAGTTATTAATATGCGAGCAGAGACTTTAACGTTTGATCGTCTTCCTTTATTTGTGTGAAGTGTTTTTATTACTGTAATCTTACTTTTACTATCTCTACCTGTATTAGCTGGAGCTATTACAATATTACTAACAGATCGAAATTTGAATACATCATTTTTTGACCCAACTGGAGGAGGTGATCC

>SDa28 [organism=Balanus glandula] [lat-lon=32.1331,-116.8882] [collection-date=2016-06-22] [mol_type=genomic DNA] [country=USA: North Pacific Ocean, California] [db_xref=taxon:110520]

TTATCGAGAAATATCGCCCATTCAGGAGCATCAGTAGATTTATCTATTTTTTCTCTCCATTTAGCTGGAGCTTCATCTATTCTTGGGGCCATTAATTTTATATCAACAGTTATTAATATGCGAGCAGAGACTTTAACGTTTGATCGTCTTCCTTTATTTGTGTGAAGTGTTTTTATTACTGTAATCTTACTTTTACTATCTCTACCTGTATTAGCTGGAGCTATTACAATATTACTAACAGATCGAAATTTGAATACATCATTTTTTGACCCAACTGGAGGAGGTGATCCGATTTTATACCAAC

>SDa29 [organism=Balanus glandula] [lat-lon=32.1331,-116.8882] [collection-date=2016-06-22] [mol_type=genomic DNA] [country=USA: North Pacific Ocean, California] [db_xref=taxon:110520]

TTTAGCTGGAGCTTCATCTATTCTTGGGGCCATTAATTTTATATCAACAGTTATTAATATGCGAGCAGAGACTTTAACGTTTGATCGTCTTCCTTTATTTGTGTGAAGTGTTTTTATTACTGTAATCTTACTTTTACTATCTCTACCTGTATTAGCTGGAGCTATTACAATATTACTAACAGATCGAAATTTGAATACATCA

>SDa30 [organism=Balanus glandula] [lat-lon=32.1331,-116.8882] [collection-date=2016-06-22] [mol_type=genomic DNA] [country=USA: North Pacific Ocean, California] [db_xref=taxon:110520]

TTTATCGAGAAATATCGCCCATTCAGGAGCATCGGTAGATTTATCTATTTTTTCTCTCCATTTAGCTGGAGCTTCATCTATTCTTGGGGCCATTAATTTTATATCTACAGTTATTAATATGCGAGCAGAGACTTTAACGTTTGATCGTCTTCCTTTATTTGTGTGAAGTGTTTTTATTACTGTAATCTTACTTTTACTATCTCTACCTGTATTAGCTGGAGCTATTACAATATTACTAACAGATCGAAATTTGAATACATCATTTTTTGACCCAACTGGAGGAGGTGATCCGATTTTATACCAACACTTATTC

>SDa32 [organism=Balanus glandula] [lat-lon=32.1331,-116.8882] [collection-date=2016-06-22] [mol_type=genomic DNA] [country=USA: North Pacific Ocean, California] [db_xref=taxon:110520]

TTTATCGAGAAATATCGCCCATTCAGGAGCATCGGTAGATTTATCTATTTTTTCTCTCCATTTAGCTGGAGCTTCATCTATTCTTGGGGCTATTAATTTTATATCAACAGTTATTAATATGCGAGCAGAGACTTTAACGTTTGATCGTCTTCCTTTATTTGTGTGAAGTGTTTTTATTACTGTAATCTTACTTTTACTATCTCTACCTGTATTAGCTGGAGCTATTACAATATTACTAACAGATCGAAATTTGAATACATCATTTTTTGACCCAACTGGAGGAGGTGATCCTATTTTATACCAACACTTATTC

>SDa33 [organism=Balanus glandula] [lat-lon=32.1331,-116.8882] [collection-date=2016-06-22] [mol_type=genomic DNA] [country=USA: North Pacific Ocean, California] [db_xref=taxon:110520]

TTTATCGAGAAATATCGCCCATTCAGGAGCATCGGTAGATTTATCTATTTTTTCTCTCCACTTAGCTGGAGCTTCATCTATTCTTGGGGCCATTAATTTTATATCAACAGTTATTAATATGCGAGCAGAGACTTTAACGTTTGATCGTCTTCCTTTATTTGTGTGAAGTGTTTTTATTACTGTAATCTTACTTTTACTATCTCTACCTGTATTAGCTGGAGCTATTACAATATTACTAACAGATCGAAATTTGAATACATCATTTTTCGACCCAACTGGAGGAGGTGATCCGATTTTATACCAACACTTATTC

>SDa34 [organism=Balanus glandula] [lat-lon=32.1331,-116.8882] [collection-date=2016-06-22] [mol_type=genomic DNA] [country=USA: North Pacific Ocean, California] [db_xref=taxon:110520]

ATCGAGAAATATCGCCCATTCAGGAGCATCGGTAGATTTATCTATTTTTTCTCTCCATTTAGCTGGAGCTTCATCTATTCTTGGGGCCATTAATTTTATATCAACAGTTATTAATATGCGAGCAGAGACTTTAACGTTTGATCGTCTTCCTTTATTTGTGTGAAGTGTTTTTATTACTGTAATCTTACTTTTACTATCTCTACCTGTATTAGCTGGAGCTATTACAATATTACTAACAGATCGAAATTTGAATACATCATTTTTTGACCCAACTGGAGGAGGTGATCCGATTTTATACCAACACTTATTC

>SDa35 [organism=Pollicipes polymerus] [lat-lon=32.8694,-117.2535] [collection-date=2016-05-09] [mol_type=genomic DNA] [country=USA: North Pacific Ocean, California] [db_xref=taxon: 36137]

GCCAGCAATATCGCACACTCAGGAGCCTCTGTAGACCTCTCTATTTTTTCATTACACTTAGCGGGAGCTTCCTCTATTTTAGGAGCTATCAACTTCATATCCACAGTAATTAATATACGAGCTGAAACTTTAACATTCGATCGTTTACCTTTATTTGTATGAAGAGTATTTGTGACGGTGATTCTTCTATTATTATCTTTACCTGTGTTAGCCGGAGCCATTACTATGCTTTTAACTGACCGGAATCTTAATACATCATTTTTCGACCCTACAGGGGGAGGAGACCCTATTTTATACCAACACTTATTC

>SDa36 [organism=Pollicipes polymerus] [lat-lon=32.8694,-117.2535] [collection-date=2016-05-09] [mol_type=genomic DNA] [country=USA: North Pacific Ocean, California] [db_xref=taxon: 36137]

TAGCCAGCAATATCGCACACTCAGGAGCCTCTGTAGACCTCTCTATTTTTTCATTACACTTAGCGGGAGCTTCCTCTATTTTAGGAGCTATCAACTTCATATCCACAGTAATTAATATACGAGCTGAAACTTTAACATTCGACCGTTTACCTTTATTTGTATGAAGAGTATTTGTGACGGTGATTCTACTATTATTATCTTTACCTGTGTTAGCCGGAGCCATTACTATGCTTTTAACTGACCGGAATCTTAATACATCATTTTTCGACCCTACAGGGGGAGGAGACCCCATTTTATACCAACACTTATTC

>SDa37 [organism=Tetraclita rubescens] [lat-lon=32.8098,-117.2693] [collection-date=2016-05-07] [mol_type=genomic DNA] [country=USA: North Pacific Ocean, California] [db_xref=taxon: 311010]

GTAATATTGCCCACTCAGGAGCCTCAGTAGATTTATCTATTTTTTCCTTGCATTTAGCAGGGGCTTCTTCCATTCTAGGTGCTATTAATTTTATATCCACAGTTATTAATATACGAGCAGAAACTTTAACTTTCGATCGTCTTCCTCTTTTTGTATGAAGTGTTTTTATTACTGTAATTCTTCTTCTTTTATCTCTCCCAGTTTTAGCAGGGGCTATTACTATACTTCTAACCGATCGAAATCTTAATACATCTTTTTTTGATCCAACTGGAGGAGGAGACCCTATTCTGTATCAACATTTATT

>SDa39 [organism=Tetraclita rubescens] [lat-lon=32.8694,-117.2535] [collection-date=2016-06-20] [mol_type=genomic DNA] [country=USA: North Pacific Ocean, California] [db_xref=taxon: 311010]

TTGCATTTAGCAGGAGCTTCTTCCATTCTAGGTGCTATTAATTTTATATCCACAGTTATTAATATACGAGCAGAAACTTTAACTTTTGATCGTCTTCCTCTTTTTGTATGAAGTGTTTTTATTACTGTAATTCTTCTTCTTTTATCTCTCCCAGTTTTAGCAGGGGCTATTACTATACTTCTAACCGATCGAAATCTTAATACATC

>SDa40 [organism=Tetraclita rubescens] [lat-lon=32.8694,-117.2535] [collection-date=2016-06-20] [mol_type=genomic DNA] [country=USA: North Pacific Ocean, California] [db_xref=taxon: 311010]

TTGCATTTAGCAGGAGCTTCTTCCATTCTAGGTGCTATTAATTTTATATCCACAGTTATTAATATACGAGCAGAAACTTTAACTTTCGATCGTCTTCCTCTTTTTGTATGAAGTGTTTTTATTACTGTAATTCTTCTTCTTTTATCTCTCCCAGTTTTAGCAGGGGCTATTACTATACTTCTAACCGATCGAAATCTTAATACAT

>SDa41 [organism=Tetraclita rubescens] [lat-lon=32.8694,-117.2535] [collection-date=2016-06-20] [mol_type=genomic DNA] [country=USA: North Pacific Ocean, California] [db_xref=taxon: 311010]

CCTTGCATTTAGCGGGAGCTTCTTCCATTCTAGGTGCTATTAATTTTATATCCACAGTTATTAATATACGAGCAGAAACTTTAACTTTCGATCGTCTTCCTCTTTTTGTATGAAGTGTTTTTATTACTGTAATTCTTCTTCTTTTATCTCTCCCAGTTTTAGCAGGGGCTATTACTATACTTCTAACCGATCGAAATCTTAATACATC

>SDa42 [organism=Tetraclita rubescens] [lat-lon=32.1331,-116.8882] [collection-date=2016-06-22] [mol_type=genomic DNA] [country=USA: North Pacific Ocean, California] [db_xref=taxon: 311010]

AGAAATATTGCCCACTCAGGAGCCTCAGTAGATTTATCTATTTTTTCCTTGCATTTAGCAGGAGCTTCCTCCATTCTAGGTGCTATTAATTTTATATCCACAGTTATCAATATACGAGCAGAAACTTTAACTTTCGATCGTCTTCCTCTTTTTGTATGAAGTGTTTTTATTACTGTAATTCTTCTTCTTTTATCTCTCCCAGTTTTAGCAGGGGCTATTACTATACTTCTAACCGATCGAAATCTTAATACATCTTTTTTTGATCCAACTGGAGGAGGAGACCCTATTCTGTATCAACATTTATT

>SDa43 [organism=Megabalanus rosa] [lat-lon=32.8694,-117.2535] [collection-date=2016-06-20] [mol_type=genomic DNA] [country=USA: North Pacific Ocean, California] [db_xref=taxon: 6680]

TCTGTAGATTTATCTATTTTTTCTTTACACTTAGCTGGAGCATCCTCAATTCTAGGTGCAATTAATTTTATGTCTACAGTTATTAACATACGAGCAGAAACTTTAACATTTGACCGTCTACCTTTGTTCGTGTGAAGAGTTTTCATTACTGTTATTTTACTATTACTTTCCTTACCAGTTTTAGCAGGTGCAATTACTATGTTGTTAACTGACCGTAATCTTAACACCTCATTCTTTGACCCTACAGGTGGAGGAG

>SDa44 [organism=Megabalanus rosa] [lat-lon=32.8694,-117.2535] [collection-date=2016-06-20] [mol_type=genomic DNA] [country=USA: North Pacific Ocean, California] [db_xref=taxon: 6680]

CTTTACACTTAGCTGGAGCATCCTCAATTCTAGGTGCAATTAATTTTATATCTACAGTTATTAACATACGAGCAGAAACTTTAACATTTGACCGTCTACCTTTGTTCGTGTGAAGAGTTTTCATTACTGTTATTTTACTATTACTTTCCTTACCAGTTTTAGCAGGTGCAATTACTATGTTGTTAACTGACCGTAATCTTAACACCTCA

>SDa45 [organism=Balanoidea sp. MM-2014] [lat-lon=32.1331,-116.8882] [collection-date=2016-06-22] [mol_type=genomic DNA] [country=USA: North Pacific Ocean, California] [db_xref=taxon: 1486016]

AATATTGCCCACTCAGGTGCCTCTGTTGATCTATCAATTTTTTCTTTACACTTAGCTGGAGCTTCATCGATTTTAGGAGCAATTAACTTTATATCGACTGTAATTAATATGCGAGCAGAGACTTTAACGTTTGATCGTTTACCTTTATTTGTATGAAGTGTATTTATTACTGTAATTTTATTATTACTTTCATTACCTGTACTTGCTGGAGCTATTACTATATTATTAACTGATCGAAACTTAAATACATCTTTCTTTGATCC

>SDa46 [organism=Balanoidea sp. MM-2014] [lat-lon=32.1331,-116.8882] [collection-date=2016-06-20] [mol_type=genomic DNA] [country=USA: North Pacific Ocean, California] [db_xref=taxon: 1486016]

CCTCTGTTGATCTATCAATTTTTTCTTTACACTTAGCTGGAGCTTCATCGATTTTAGGAGCAATTAACTTTATATCGACTGTAATTAATATGCGAGCAGAGACTTTAACGTTTGATCGTTTACCTTTATTTGTATGAAGTGTATTTATTACTGTAATTTTATTATTACTTTCATTACCTGTACTTGCTGGAGCTATTACTATATTATTAACTGATCGAAACTTAAATACATCTTTCTTTGATCCCACA

>SDa47 [organism=Balanoidea sp. MM-2014] [lat-lon=32.1331,-116.8882] [collection-date=2016-06-22] [mol_type=genomic DNA] [country=USA: North Pacific Ocean, California] [db_xref=taxon: 1486016]

GCCTCTGTTGATCTATCAATTTTTTCTTTACACTTAGCTGGAGCTTCATCGATTTTAGGAGCAATTAACTTTATATCGACTGTAATTAATATGCGAGCAGAGACTTTAACGTTTGATCGTTTACCTTTATTTGTATGAAGTGTATTTATTACTGTAATTTTATTATTACTTTCATTACCTGTACTTGCTGGAGCTATTACTATATTATTAACTGATCGAAACTTAAATACATCTTTCTTTGATCCCACAGGAGGAGGTGATCCAATTCTTTATCAACATTTATTC
