# Supplementary figures and images for: Diversity and distribution of nearshore barnacle cyprids in southern California through the 2015–16 El Niño
Source: PeerJ. 2019 Jul 1;7:e7186. doi: 10.7717/peerj.7186 (PMC6610546; doi:10.7717/peerj.7186)

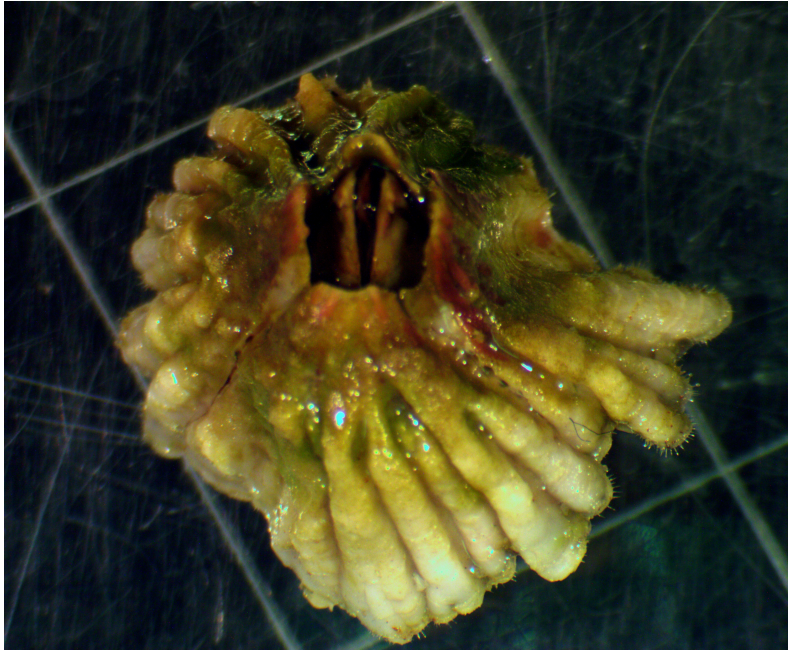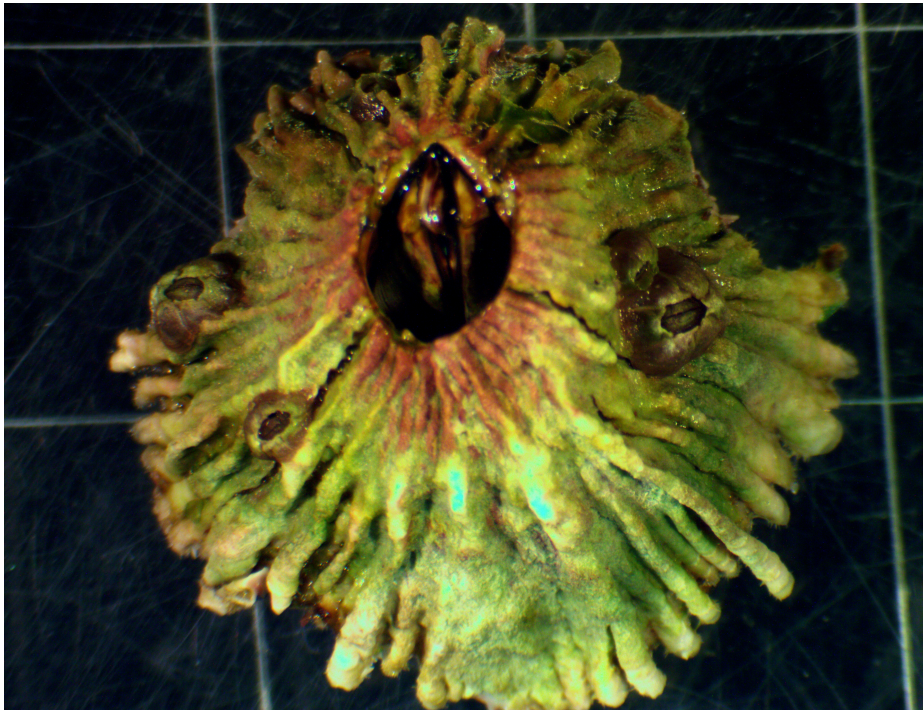

Supplement: Supplemental Information 3 — Photos of 2 of the unknown Balanoidea sp. adult barnacles collected in the intertidal at Dike Rock, La Jolla, California, USA and Alisitos, Baja California, Mexico. [file peerj-07-7186-s003.pdf]
